# Phylogenetic comparison and splice site conservation of eukaryotic U1 snRNP-specific U1-70K gene family

**DOI:** 10.1038/s41598-021-91693-3

**Published:** 2021-06-17

**Authors:** Tao Fan, Yu-Zhen Zhao, Jing-Fang Yang, Qin-Lai Liu, Yuan Tian, Das Debatosh, Ying-Gao Liu, Jianhua Zhang, Chen Chen, Mo-Xian Chen, Shao-Ming Zhou

**Affiliations:** 1 Division of Gastroenterology, Shenzhen Children’s Hospital, Shenzhen, 518038 People’s Republic of China; 2 Department of Infectious Disease, Nanjing Infectious Disease Center, The Second Hospital of Nanjing, Nanjing University of Chinese Medicine, Nanjing, 210003 People’s Republic of China; 3 State Key Laboratory of Crop Biology, College of Life Science, Shandong Agricultural University, Taian, Shandong People’s Republic of China; 4 Shenzhen Research Institute, The Chinese University of Hong Kong, Shenzhen, People’s Republic of China; 5 Key Laboratory of Pesticide and Chemical Biology, Ministry of Education, College of Chemistry, Central China Normal University, Wuhan, 430079 People’s Republic of China; 6 School of Basic Medicine, Shandong First Medical University and Shandong Academy of Medical Sciences, Qingdao, People’s Republic of China; 7 Department of Biology, Hong Kong Baptist University, and State Key Laboratory of Agrobiotechnology, The Chinese University of Hong Kong, Shatin, Hong Kong

**Keywords:** Evolution, Genetics

## Abstract

Eukaryotic cells can expand their coding ability by using their splicing machinery, spliceosome, to process precursor mRNA (pre-mRNA) into mature messenger RNA. The mega-macromolecular spliceosome contains multiple subcomplexes, referred to as small nuclear ribonucleoproteins (snRNPs). Among these, U1 snRNP and its central component, U1-70K, are crucial for splice site recognition during early spliceosome assembly. The human U1-70K has been linked to several types of human autoimmune and neurodegenerative diseases. However, its phylogenetic relationship has been seldom reported. To this end, we carried out a systemic analysis of 95 animal *U1-70K* genes and compare these proteins to their yeast and plant counterparts. Analysis of their gene and protein structures, expression patterns and splicing conservation suggest that animal U1-70Ks are conserved in their molecular function, and may play essential role in cancers and juvenile development. In particular, animal *U1-70Ks* display unique characteristics of single copy number and a splicing isoform with truncated C-terminal, suggesting the specific role of these U1-70Ks in animal kingdom. In summary, our results provide phylogenetic overview of U1-70K gene family in vertebrates. In silico analyses conducted in this work will act as a reference for future functional studies of this crucial U1 splicing factor in animal kingdom.

## Introduction

Precursor-mRNA splicing is a crucial eukaryotic molecular mechanism which was discovered nearly half a century ago^1^. It consists of removal of introns present between exon sequences by a two-step trans-esterification reaction^2^. This sophisticated mechanism is carried out by spliceosome, a mega-molecular protein complex that is recruited co-transcriptionally^4,5^. The spliceosome itself contains several subcomplexes, called small nuclear ribonucleoprotein particles (snRNPs) including U1, U2, U4, U5, U6, U11 and U12. Each snRNP is further composed of common and RNP-specific proteins on a structural snRNA^6^. Furthermore, snRNP subcomplexes are initially assembled in a tightly controlled manner for the recognition of splice-specific sequences such as branch point sequence, 5′ and 3′ splice sites^3,7^. These sequences are recognized by special snRNPs such as U1 and U2 snRNP or splicing factors such as splicing factor 1 (SF1) and U2 snRNP auxiliary factors (U2AFs)^8,9^. U1 snRNP is the first subcomplex during spliceosome assembly and is composed of 8–9 Sm common core proteins and a number of U1-specific proteins (U1A, U1C and U1-70K etc.) in human and yeast^10^. Specifically, this subcomplex is responsible for the 5′ splice site selection in both constitutive and alternative splicing (AS)^3,11^. Additional role of U1 snRNP has been found in other RNA processing mechanisms such as 3′-end polyadenylation and cleavage^12,13^.

Initial characterization of this snRNP suggested its links to human pathogenesis and autoimmune diseases with unknown etiology, making it a potential diagnostic biomarker. For example, U1 snRNP has been identified as a putative target in autoimmune disorder called mixed connective tissue disease (MCTD). Autoantibodies are produced in patients suffering from MCTD especially against a 40 kDa form of U1-70K protein which is cleaved by caspase-3 during apoptosis^14^. Further studies indicate that the interaction among immune cells, U1-70K and its binding backbone, U1 snRNA might be the primary cause of inflammation and tissue injury in MCTD patients^15^. In other instances, autoantibodies recognizing U1 snRNPs are frequently detected in at least 30–40% of patients with systemic lupus erythematosus (SLE) and other rheumatic diseases^16^. In particular, RNP1 motif region (131–151 aa) of U1-70K has been demonstrated to play an important role to prevent intermolecular T-B cell diversification^17^. A synthesized peptide analogue phosphorylated on Ser140 was shown to be successful in treating SLE patients by modulating T cell response and altering the autophagy pathway^18–21^. In addition, a significant association between the co-existence of antibodies to cytomegalovirus (CMV) and snRNPs has been observed in SLE patients^22^, suggesting the crosstalk between CMV and autoimmunity of SLE. Besides these autoimmune diseases, neurodegenerative diseases such as Alzheimer’s disease (AD) may result from splicing defects due to disruption in U1 snRNPs. Pathological evidence of human brain-insoluble proteome has demonstrated the unique aggregation pattern of U1 snRNPs in AD diseased neuronal cells^23^, causing global splicing defects in the early stages of AD in the affected patients. The splicing pattern of its downstream target, Presenilin-2 (PS2) is affected by an exon skipping event at exon 5, an event constantly detected in AD patients^24^. Intriguingly, aggregated proteolytic products of U1-70K have been implicated in neuronal toxicity in AD as well. Furthermore, the molecular mechanism that links U1 snRNP to other diseases such as congenital myasthenic syndrome (CMS), has been reported recently^25^. Interestingly, the disruption of U1 snRNP assembly is thought to be of potential application to regulate the replication of Human Immunodeficiency Virus (HIV-1) suggesting positive applications of studying the spliceosome assembly components^26^. In summary, U1snRNPs such as U1-70K can be considered to be a crucial regulator of a variety of human diseases and the study of its phylogeny and splicing pattern may help us to identify the potential molecular function of this splicing factor.

To this end, we carried out a phylogenetic analysis of 95 species/strains available on the Ensemble database (http://asia.ensembl.org/index.html). Subsequently, additional in silico analysis was performed to elucidate the conservation of gene and protein structures and discuss U1-70Kspatio-temporal expression and conserved splicing patterns. The outcome of this study should provide readers with a background information of this gene family which could be used for further functional investigations.

## Materials and methods

### Sequence identification of the animal U1-70K proteins

The U1-70K protein sequence (ENSP00000472998.1) of *Homo sapiens* was used as a query to carry out BLASTp search with *e*-value cutoff = 1*e*^−10^ against all available animal genome sequences from Ensembl database http://asia.ensembl.org/index.html) as described previously^27,28^. The obtained sequences consisting of both PF00076.22 (RNA recognition motif, RRM_1) and PF12220.8 (U1 small nuclear ribonucleoprotein of 70 kDa MW N terminal, U1snRNP70_N) protein domains were further screened by HMMER (https://www.ebi.ac.uk/Tools/hmmer/search/phmmer). Finally, a total of 95 U1-70K protein sequences from 95 animal species/strains (Table S1) were selected for further analysis.

### Phylogenetic analysis of *U1-70K* gene family in animals

The amino acid sequences of 95 *U1-70K* genes mentioned above were used for the construction of phylogenetic tree. The longest protein-coding sequence of each U1-70K was chosen for genes with multiple transcript isoforms. Subsequently, Muscle v3.8 was used for multiple sequence alignment of all selected *U1-70K* sequences^29^ and a rooted phylogenetic tree was built by using maximum likelihood implemented in PhyML v3.037^30^. The final visualization of phylogenetic trees was obtained with FigTree v1.4.3.38^31^. Plant U1-70K sequences (Chen et al. 2019) were used to construct a larger tree against animal and yeast U1-70K sequences for cross-kingdom comparison.

### Investigation of protein domain, gene structure and conserved motifs

Protein domains were predicted by HMMER database and the exon–intron structures of all genes were downloaded and reconstructed from Ensembl database. The gene coding sequence (CDS) and protein sequences of all genes were used as input into Multiple Em for Motif Elicitation (MEME) (http://meme-suite.org/tools/meme)^32^, to obtain the top 10 conserved motifs. Arabidopsis U1-70K (At3g50670) was used for comparison against human and yeast U1-70Ks.

### Construction of protein interaction networks

Protein–protein interaction networks of U1-70K proteins from *Homo sapiens* (ENSP00000472998.1), *Mus musculus* strain C57BL/6NJ (MGP_C57BL6NJ_P0084319) and *Saccharomyces cerevisiae* (YIL061C) were analyzed on STRING database (https://string-db.org/). Finally, top 10 interaction partners of each U1-70K protein was presented in the form of an interaction network.

### Homology modeling and amino acid conservation estimation

Amino acid conservation scores were calculated utilizing Maximum Likelihood (ML) method employed at ConSurf web server (https://consurf.tau.ac.il/) after curating the sequences with gaps^33^. Multiple sequence alignment and structural data were provided as input attributes. The crystal structure of human U1-70K protein (PDBID: 6QX9) was downloaded from PDB database^34^. Homology modelling of *Arabidopsis thaliana* U1 SNRNP70 was conducted by using it (identity: 44.33%) as a template on the Swiss-Model server^35^. Figures were drawn with default PyMOL settings^36^.

### Conserved AS profile analysis and identification of conserved splice sites

All available splicing isoforms of animal *U1-70K* genes were obtained again from Ensembl database. Selected splice junction sequences (i.e. 31-bp in total, 15-bp exon sequence and 16-bp intron sequence) were further examined using BLAST. Consensus sequences at representative splice sites were analyzed and visually represented by using Weblogo v3.0 (https://weblogo.berkeley.edu/logo.cgi).

### Expression analysis of U1-70Ks from available microarray datasets

Expression data for selected *U1-70Ks* were download from Expression Atlas (https://www.ebi.ac.uk/gxa/home). The raw data was reorganized and presented as heatmaps by using online BAR HeatMapper Plus software (http://bar.utoronto.ca/ntools/cgi-bin/ntools_heatmapper_plus.cgi).

## Results

### Identification of animal *U1-70K* genes for phylogenetic tree construction

To identify putative *U1-70K* genes throughout animal species, amino acid sequences of Human U1-70K (*Homo sapiens*, ENSP00000472998.1) was used to perform BLAST search against all available sequences in Ensembl database. All positive hits were then subjected to online software HMMER for protein domain analysis and prediction. As a result, a total of 95 *U1-70K* genes were identified from 95 animal species (Table S1), including 23 primates, 36 rodents and lagomorphs, 17 other mammals, 14 other vertebrates and 5 other species (*Ciona intestinalis*, *Ciona savignyi*, *Caenorhabditis elegans*, *Drosophila melanogaster* and *Saccharomyces cerevisiae*). Intriguingly, compared to 115 sequences identified from 67 plant species, especially ten copies of U1-70K genes identified from *Triticum aestivum* (Fig. S2), animal U1-70Ks exhibit exclusively one copy in each species/strain with a high sequence similarity, suggesting its conserved function in animal kingdom.

Expectedly, phylogeny of *U1-70K* genes largely correlates with the evolutionary relationships among species. In order to obtain a more comprehensive phylogenetic relationship of *U1-70K* gene family in animals, a rooted phylogenetic tree of animal *U1-70K* family was subsequently constructed based on multiple sequence alignment of above selected 95 animal U1-70K protein sequences (Fig. S1). The constructed phylogenetic tree was associated with overall median to high bootstrap values represented by a color gradient. The tree can be divided into three major clades. In particular, one small clade with a longer branch length and consisting of five other species (purple sector) formed the basal part of this tree, indicating the distant relation of this clade with other animal *U1-70K* genes. Furthermore, a majority of vertebrates including primates (e.g.* Homo sapiens*, red sector), rodents and lagomorphs (e.g.* Mus musculus* and *Rattus norvegicus*, green sector), other mammals (orange sector) and one species from the category of other vertebrates (Chinese softshell turtle), were clustered into a second clade (Fig. S1). The remaining vertebrates (pink sector), covering all genes from birds, reptiles and fish species, formed a paraphyletic sister clade to the second clade (Fig. S1). Hence, genes from the phylogenetically related animal species tend to cluster together in the tree. For example, *U1-70K* genes from the Primates species including *Homo sapiens* and its close relatives fell into one distinct monophyletic group. However, an exception was found in the subclade rodents and lagomorphs (green sector), the U1-70K (ENSPSIP00000017206.1) of *Pelodiscus sinensis* (Chinese softshell turtle) was grouped here, but not with other vertebrates (pink sector). Given that the BS value of this species is relatively low, it has a possibility that this grouping may be a result of instability in the phylogenetic tree.

### Protein domain/motif analysis

**Figure 1:**
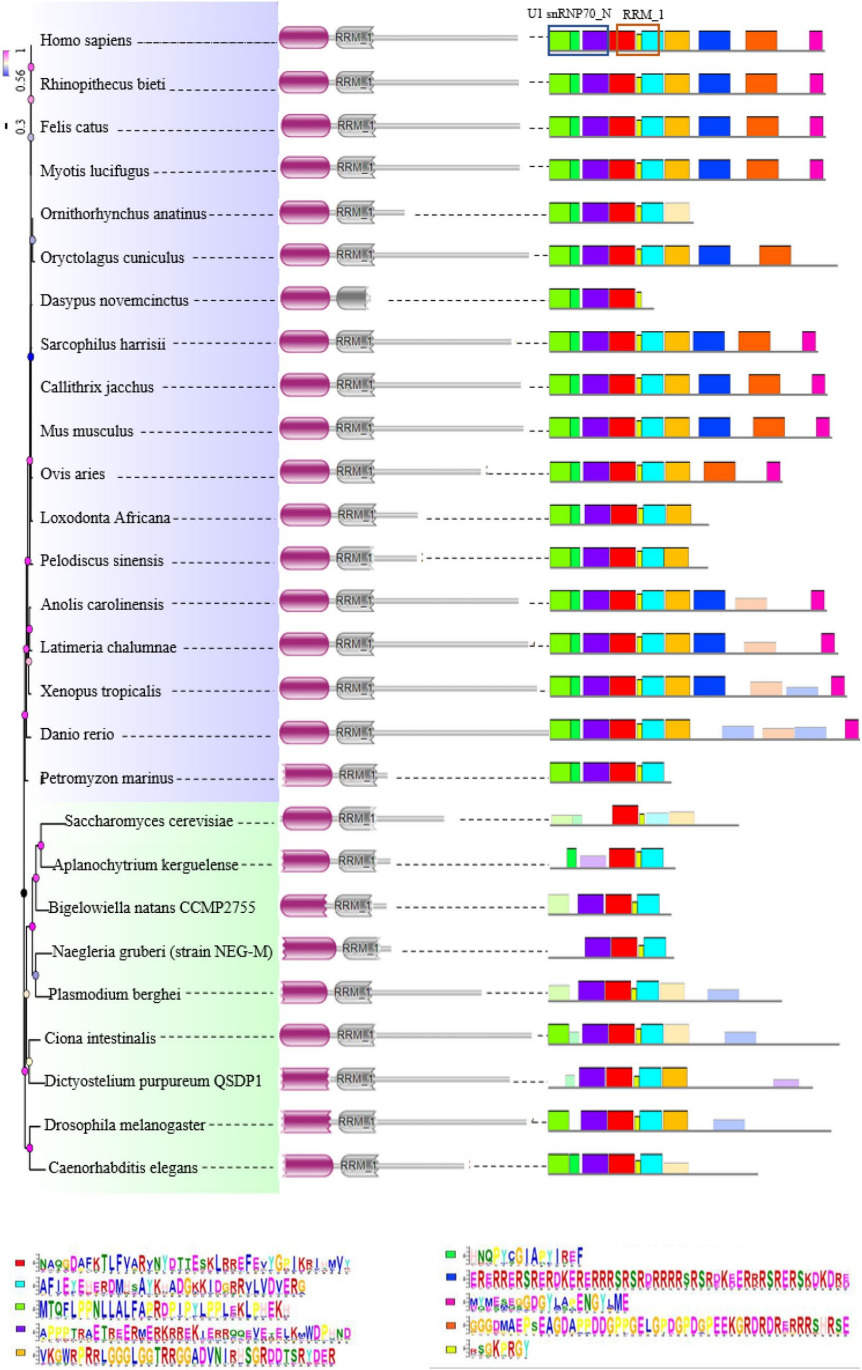
Protein motif analysis of representative animal U1-70Ks. The phylogenetic relationship is listed below. Protein regions predicted by online software HMMER are listed on the middle panel. Conserved motifs analyzed by MEME online tool are listed on the right panel. Top ten conserved motifs are represented by different colored boxes. For conserved motifs, the height of a box indicates the significance of the site (i.e. taller boxes are more significant). The correlation between major protein domains (middle panel) and conserved motifs (right panel) are shown in blue and red frames for U1 snRNP70_N and RRM_1 region, respectively.

**Figure 2:**
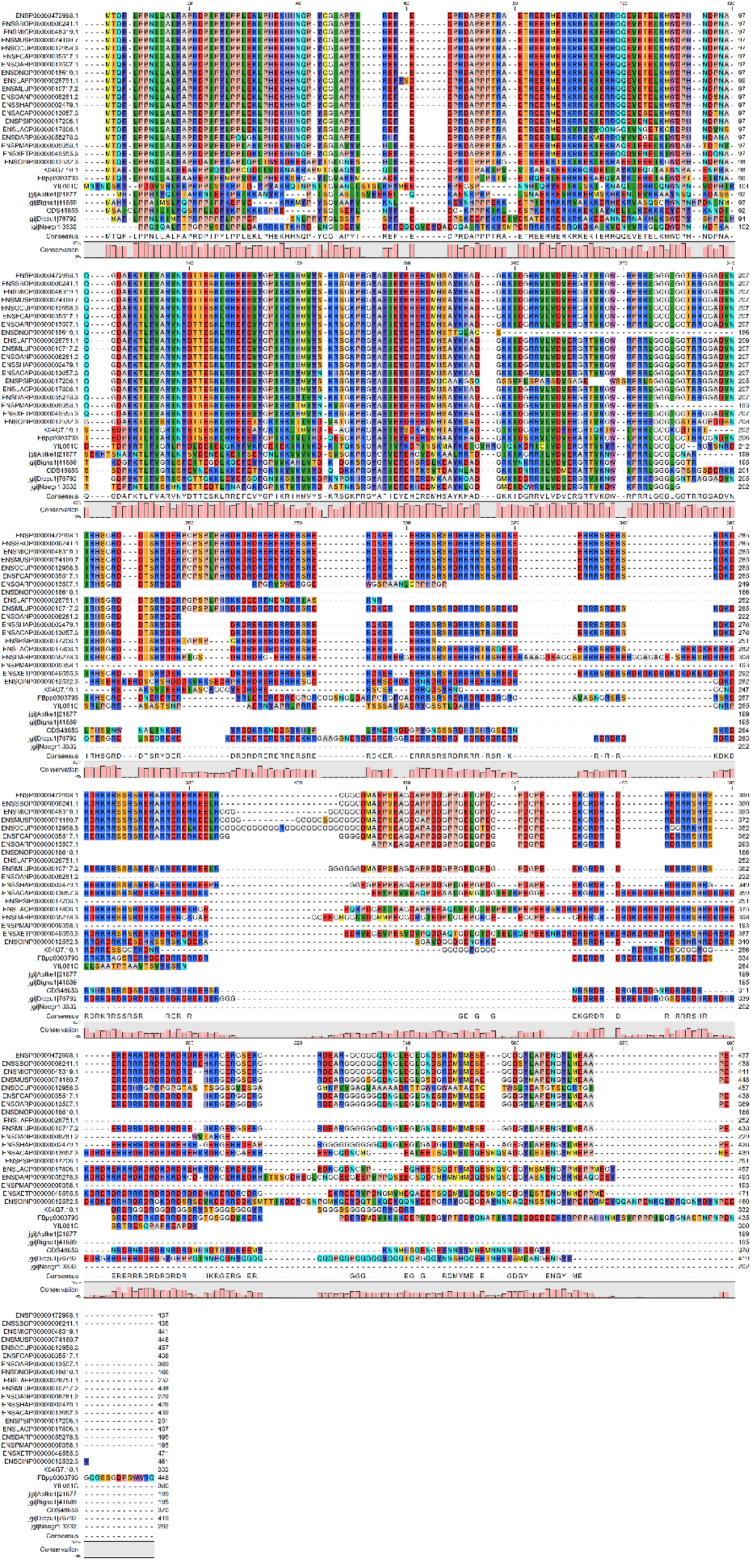
Multiple sequence alignment by using 27 representative animal U1-70K sequences for phylogenetic tree construction.

In order to further investigate the conservation of animal U1-70Ks, protein domains and conserved motifs were subjected to detailed analysis. Totally 27 representative animal species of U1-70Ks were further aligned to construct a phylogenetic tree (Figs. 1 and 2). According to the results predicted by online software HMMER (Fig. 1, middle panel), all U1-70K proteins selected here contained both U1snRNP70_N and RNA recognition motif (RRM, RBD, or RNP domain). Similar to the phylogeny of 95 animal U1-70Ks protein sequences (Fig. S2), the length of all identified U1-70K proteins were characterized in a range of 159 to 499 amino acids. Most of the U1-70K proteins were approximately 440 amino acids in length (Table S2). Specifically, the size of both conserved RRM_1 and U1snRNP70_N domain were strictly maintained at ~ 70 and ~ 90 amino acids, respectively. However, low complexity and coiled coil regions of some U1-70K proteins were predicted to be longer than 160 amino acids, possibly due to the incorporation of unknown domains.

Multiple Em for Motif Elicitation (MEME) online tool was used to analyze the conserved motifs in animal U1-70K proteins (Fig. 1 and Fig. S2, right panel). Top ten conserved motifs were chosen and illustrated in colored boxes, covering the entire identified U1-70K protein sequences. Proteins from vertebrates, except those with significantly truncated sequences, normally contained ten conserved motifs. For example, U1-70Ks of fish and birds contain six to seven motifs, whereas shorter sequences, such as ENSHGLP00100002115.1, contained only four conserved motifs. In contrast, U1-70K proteins in the category of other species contained only zero to five motifs. Despite the differences in motif numbers, the orders of the motifs remained the same across all U1-70Ks. Expectedly, gaps between conserved motifs were observed in proteins with a larger size, such as ENSMUST00000074575.10 of mouse, indicating acquisition of novel sequences between conserved motifs. The U1snRNP70_N and RRM_1 domain was present in the first three and the next two motifs, respectively, at the N-terminal of U1-70Ks (Fig. 1 and Fig.  S2, right panel). In contrast, the C-terminal of U1-70Ks were highly variable. For example, U1-70Ks of *Dasypus novemcinctus* (ENSDNOP00000018610.1), *Ictidomys tridecemlineatus* (ENSSTOP00000012536.3), *Equus caballus* (ENSECAP00000011711.1), *Heterocephalus glaber* (ENSHGLP00100002115.1) and *Sus scrofa* (ENSSSCP00000043949.1) were truncated at their C-terminal to yield a short protein product (159–202 a.a. in length), probably due to incomplete annotation of their genomes. In addition, U1-70Ks of sector ‘other vertebrates’ and ‘other species’ were shown less conservation at their C-terminal, imply potential function diversification.

### Homology modeling, protein conservation and interaction network of U1-70Ks

As U1-70K is a central component of U1 snRNP complex, understanding of its conserved structure across animal species is crucial for future comparative biochemical and functional studies. The possibilities of amino acid residues with highest frequency in each position ranged from 86.52 to 100%, indicating the high conservation of animal U1-70Ks. The complex crystal structure of human U1-70K and RNA was presented here (Fig. 2). There were nine residues (Arg71, Lys74, Asn96, Tyr112, Arg139, Arg144, Tyr146, Thr199, and Arg200) in U1-70K may imply the importance of electrostatic interaction for RNA binding. Among these residues, Asn96, Tyr112, and Tyr146 were highly conserved at ConSurf Grade 9. Arg71 (98.88%) and Arg144 (97.75%) were conserved at ConSurf Grade 7. Thr199 (97.59%) and Arg200 (97.59%) were conserved at ConSurf Grade 5. Lys74 (98.88%) and Arg139 (92.135%) were conserved at ConSurf Grade 6 and ConSurf Grade 1.

To compare the structures of U1-70K in plants and animals, homology modeling of *Arabidopsis thaliana* U1-70K was performed based on the crystal structure of human U1-70K (PDBID: 6QX9) (Fig. S3), to perform conservation analysis. The structures of *Arabidopsis thaliana* and human U1-70Ks were superimposed and colored according to ConSurf Grade (Fig. S3 and Table S3) and the corresponding phylogenetic relationship and multiple sequence alignment of plant and animal U1-70Ks is shown in Figs. S4 and S5. The overall percentage of conservation of plant U1-70K was lower than that of animal. The possibilities of residues with highest frequency in each position ranged from 26.97 to 100%.

Marked differences were observed between the nine important residues of animals and plants, as follows: His106 (Arg71), Arg109 (Lys74), Leu174 (Arg139), Lys179 (Arg144), and Ser234 (Thr199). For plants, Tyr181 (animal: Tyr146, 100%) and Arg235 (animal: Arg200, 98.84%) were conserved at ConSurf Grade 9; His106 (animal: Arg71, 68.54%) and Asn131 (animal: Asn96, 83.15%) were conserved at ConSurf Grade 4; Tyr147 (animal: Tyr112, 98.88%), Lys179 (animal: Arg144, Max in plant: Arg, 76.40%), Arg109 (animal: Lys74, 71.91%), Ser234 (animal: Thr199, Max in plant: Thr, 60.23%) and Leu174 (animal: Arg139, Max in plant: Glu, 32.58%) were conserved at ConSurf Grade 8, ConSurf Grade 7, ConSurf Grade 6, ConSurf Grade 3 and ConSurf Grade 1. Among them, the residues of animal Arg71 and Arg139 showed a complete difference in comparison to the plant residues at the same positions. The different residues of plant may reduce the binding affinity to RNA. In contrast, Tyr146/181, Arg200/235, and Tyr112/147 of animals and plants, respectively, were highly conserved, which may play important role in RNA binding. However, further investigation needs to be carried out to validate these hypotheses.

**Figure 3:**
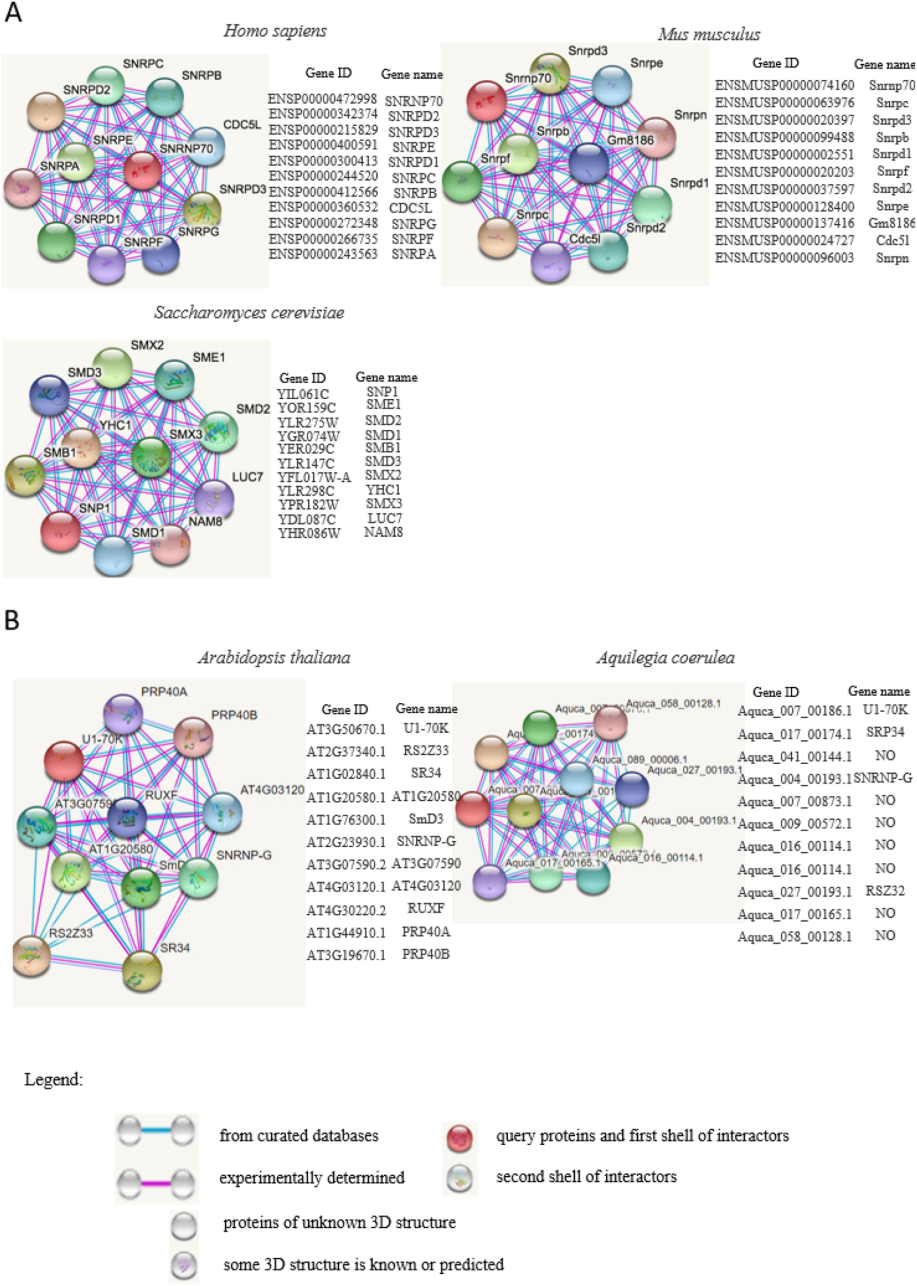
Protein–protein interaction networks of representative animal species (**A**) and plant species (**B**). Known interactions, either determined by experiments (pink line) or from curated databases (blue line) are presented in protein–protein interaction networks. U1-70Ks of *Homo sapiens*, *Mus musculus*, *Saccharomyces cerevisiae*, *Arabidopsis thaliana* and *Aquilegia coerulea* are used as query protein for analysis by STRING database. Highly scored interactors are presented in the form of network diagram. Empty notes are proteins of unknown 3D structure, while filled notes are proteins with known or predicted 3D structure in current database.

Besides the conserved 3-D structure, the interaction network of U1-70K may further reveal their involvement in various biological processes. To this end, webtool STRING was used for the construction of protein interaction networks of animal U1-70Ks (Fig. 3). We chose to present highly scored protein interactors with experimental prove of their interactions with U1-70K from model organisms including two animal species (*Homo sapiens*, *Mus musculus*), yeast (*Saccharomyces cerevisiae*), and two plant species (*Arabidopsis thaliana* and *Aquilegia coerulea*) Human SNRPD2 scored 0.999 as a protein interactor of SNRNP70 (gene name of U1-70K)^37,38^. It is a Small nuclear ribonucleoprotein Sm D2 which is a core component of the spliceosomal U1, U2, U4 and U5 small nuclear ribonucleoproteins (snRNPs), the building blocks of the spliceosome. Thereby, it plays an important role in the splicing of cellular pre-mRNAs. Most spliceosomal snRNPs contain a common set of Sm proteins SNRPB, SNRPD1, SNRPD2, SNRPD3, SNRPE, SNRPF and SNRPG that assemble in a heptameric protein ring on the Sm site of the small nuclear RNA to form the core snRNP. Similarly to human SMNRPD2, Snrpd2 in mouse scored 0.995 as a protein interactor of Snrnp70 (gene name of mouse U1-70K), which is a Small nuclear ribonucleoprotein Sm D2 in mouse. SMB1 in mouse was a 0.999 scored protein interactor of SNP1 (gene name of U1-70K), which is Small nuclear ribonucleoprotein-associated protein B; Core Sm protein SmB; part of heteroheptameric complex (with Smd1p, Smd2p, Smd3p, Sme1p, Smx3p, and Smx2p) that again is a part of the spliceosomal U1, U2, U4, and U5 snRNPs^39^. It is a homolog of human SmB and SmB’ and belongs to the snRNP SmB/SmN family.

Interestingly, human and mouse share 8 out of total 11 protein interactors, whereas yeast U1-70K (YHC1) seems to bind a different batch of spliceosomal proteins. Moreover, two kind species of plants share only 2 out of total 11 protein interactors (Fig. 3B), suggesting that two species selected for analysis has a different protein network for their own U1-70Ks. However, further validation is required to reveal specific molecular function between U1-70Ks and their protein partners.

### Analysis of genomic organization and conserved motifs in animal *U1-70K* genes

After protein level, the possible conservation of gene structure and gene-motif composition at genomic level was further explored. Therefore, exon–intron organization of each animal U1-70K with the longest CDS region was downloaded from the Ensembl database and re-constructed for conserved motif analysis (Fig. S6, middle and right panels). Generally, most of the *U1-70K* genes had a 9 exon–8 intron organization and a 1300–1400 bp CDS region, suggesting conservation of constitutive splicing pattern in U1-70Ks among animal species (Table S2). However, the length of untranslated regions (UTRs) and introns was highly variable among individual genes, as long as 20,000 bp in length (for example, in MGP_SPRETEiJ_T0082830.1 of *Mus spretus* and ENSMLET00000037088.1 of *Mandrillus leucophaeus*). In addition, fewer exons could be caused by exon fusion events with no loss of conserved motifs. Specifically, ENSOANT00000008283.2 (eight exons) of *Ornithorhynchus anatinus* and ENSORLT00000021469.1 (eight exons) from *Oryzias latipes* were examples of exon fusion. On the contrary, an exon number greater than 9, could have the same number of motifs (e.g. ENSAMET00000015898 in *Ailuropoda melanoleuca*), because of exon separation. Intriguingly, proteins with larger size often had extra exons in their CDS regions with no conserved motifs predicted. One example was the 10-exon RRM_1 domain containing ENSNGAT00000027940.1 of *Nannospalax galili*. Thus, the sequence obtained from this additional 10th exon might evolve a novel function for U1-70K.

Thus, the gene structure sometimes did not correlate with the phylogenetic relationship predicted by protein sequence. However, conserved motifs identified from U1-70K cDNAs closely correlates the motifs identified using peptide sequences (Fig. 1 and Fig. S2, right panel). Nearly all *U1-70K* genes contained 10 predicted motifs, with few exceptions in the sector ‘other species’. Truncated transcripts were found in ENSPMAT00000009398.1, YIL061C, FBtr0331375 and K04G7.10.1, which lacked several continuous nucleotide motifs at either N- or C-terminus resulting from exon loss (Fig. S6, middle panel). Furthermore, multiple less-conserved motifs were detected at the 3′-end of *U1-70K* transcripts, further confirming with peptide analysis results that animal U1-70Ks varied at their C-terminal (3′ end) regions.

### Analysis of transcript isoforms and conserved splice sites

**Figure 4:**
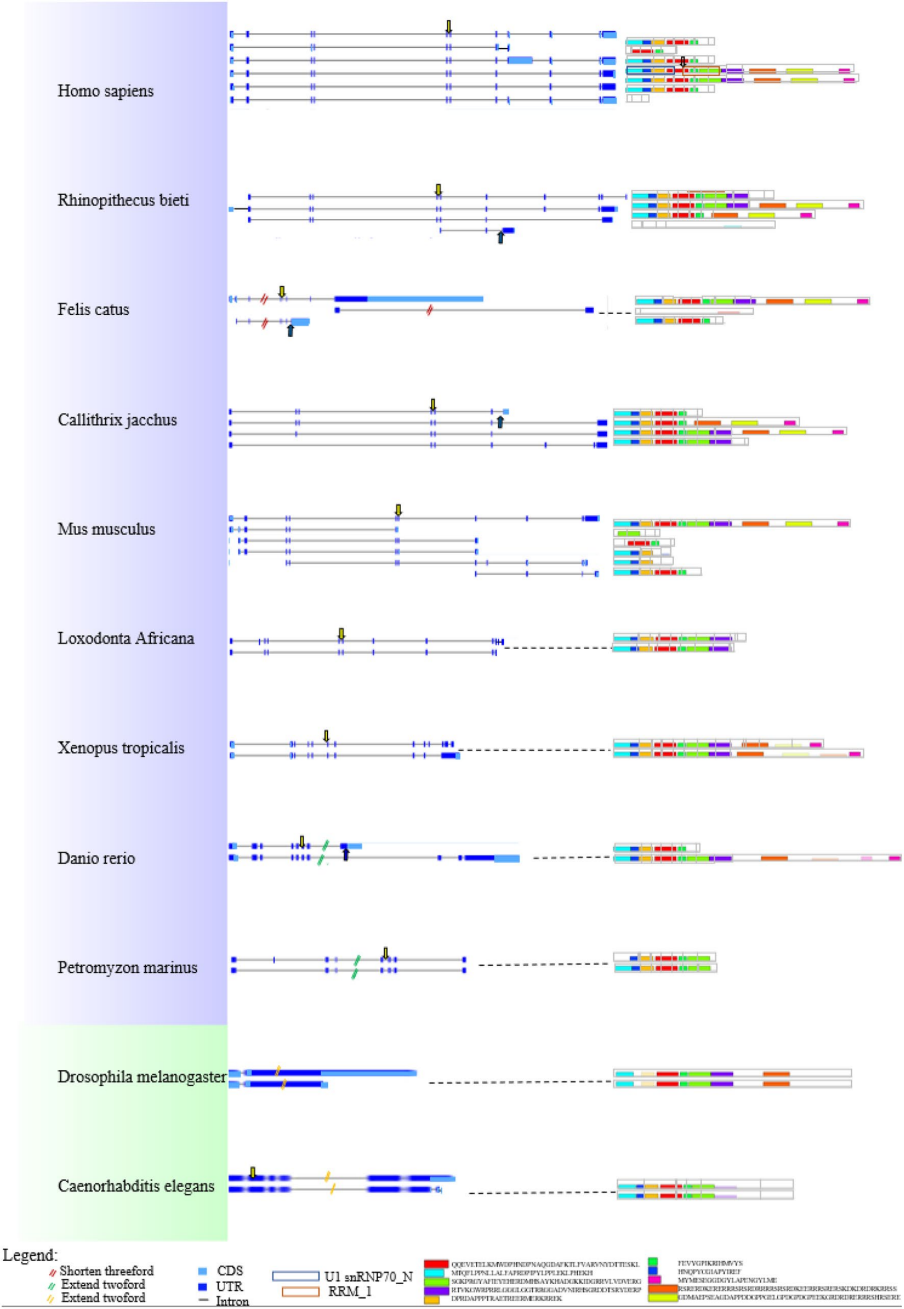
Summary of splicing isoforms for representative animal *U1-70K* genes. Transcript isoforms from 27 animal *U1-70K* genes are summarized (left and middle panel). Conserved protein motifs and sequences of potential protein products from splicing isoforms are illustrated (right panel and bottom of the figure, respectively) with additional annotation to define exon–exon boundaries (grey lines between boxes). The blue arrows indicate conserved sequences found in various species. The yellow arrows indicate the conserved splice site located in the region of RRM_1 domain with or without the detection of particular splicing events, respectively.

It has been reported that nearly all genes in human are able to undergo alternative splicing. Thus, AS analysis among various animal species could reveal the conservation of splicing patterns and splice sites among these species. After gene structure analysis, transcript level analysis was performed. For this, all available transcript isoforms of animal *U1-70K* genes were extracted from Ensembl database and displayed along with the phylogenetic relationships among selected species (Fig. S7, left and middle panels) and 27 representative animal *U1-70K* genes (Fig. 1) were presented in main text (Fig. 4). Totally, 128 splicing isoforms were obtained from 40 *U1-70K* genes and approximately 3–4 transcripts per gene. In particular, 7 and 6 isoforms were annotated for human and mouse *U1-70K* genes, respectively, having most number of isoforms obtained among the included animal species. Furthermore, comparison of conserved motifs against genomic structure (exon boundary: gray frame) of transcript isoforms was carried out (Fig. S7, right panel). Primary transcripts (the first transcript) carried the highest number of motifs, whereas alterative transcripts usually were shorter and possessed reduced number of motifs. The major AS events of U1-70Ks were identified as alternative first and last exons (AFE and ALE), leading to the generation of truncated isoforms. Few exon skipping events were also observed such as in *Petromyzon marinus*. In addition, some transcripts also displayed alternative transcription initiation and alternative polyadenylation, for example, one alternative transcript of ENSCAFT00000005969.4 from *Canis lupus familiaris*. Interestingly, animal U1-70Ks likely generated a series of truncated isoforms at their 3′-ends, potentially translating into a proteoform with either complete or shortened RRM domain (Fig. S7, right panel).

Given that a conserved splicing pattern was observed across included animal species, we further examined splice sites covering the region that can undergo alternative splicing. Interestingly, several splice sites were identified by using 31-bp flanking sequence at exon–intron junctions (Figs. S7 and S8). More specifically, two types of splice sites were characterized potentially to form different proteoforms having a variety of C-terminal sequences (Fig. S8A). The first type of splice site (indicated by yellow arrows) generated an ALE event by producing a new exon at the 3′ end of transcript, and was conserved among selected primates including *Cercocebus atys, Mandrillus leucophaeus, Macaca nemestrina, Papio anubis, Colobus angolensis palliatus, Rhinopithecus roxellana, Macaca fascicularis* and *Rhinopithecus bieti* (Fig. S8B). Furthermore, the second type of conserved splice site (as indicated by solid and hollow blue arrows) was found in three sectors such as primates, rodents/lagomorphs, and ‘other mammals’ (Fig. S8C). Intriguingly, these conserved sites were also found in *U1-70K* genes that did not have the short isoform annotation (Fig. S8D). Furthermore, the intron variable part at 5′ end flanking sequences were due to the variable sequences in sector ‘rodents/lagomorphs’ and ‘other mammals’ (Fig. S8E,F).

### Expression profile analysis of animal U1-70Ks

To further study the potential regulation of animal *U1-70K* genes in response to developmental cues or disease correlations, we analyzed the expression pattern of *U1-70K* genes from model organisms, *Homo sapiens* and *Mus musculus*. By using BAR HeatMapper Plus tool, we were able to reconstruct expression profile in the three aspects including (a) human disease (Fig. S9), (b) whole organism/tissue-specific part of human and mouse (Figs. S10 and S11) and (c) cell type and developmental stage (Figs. S12 and S13). First, human *U1-70K* gene was found to accumulate in several cancer-types such as breast cancer (breast tumor luminal, HER2 positive breast carcinoma and triple-negative breast cancer), colon cancer (colon adenocarcinoma and colon mucinous adenocarcinoma) and rectal cancer (rectal cell carcinoma and rectal mucinous adenocarcinoma) (Fig. S9). Second, *U1-70Ks* of human and mouse were highly expressed in brain tissues (Fig. S10. However, differential expression was detected between human and mouse *U1-70Ks.* Human *U1-70K* was also specifically accumulated in ovary and several glands, whereas mouse *U1-70K* gene was abundant in olfactory bulb and embryonic tissue (Figs. S10 and S11). Third, cell-type expression analysis indicated that human *U1-70K* was abundant in the mature eosinophil and plasma cells, whereas mouse *U1-70K* accumulated in astrocyte, mesodermal cell, naïve thymus-derived and T cells (Figs. S12 and S13). Developmental map suggested that human U1-70K was in high abundance at fetal stage and was down-regulated at juvenile stage (Fig. S12). In contrast, mouse U1-70K was highly expressed in adults but not in fetus (Fig. S13).

**Figure 5:**
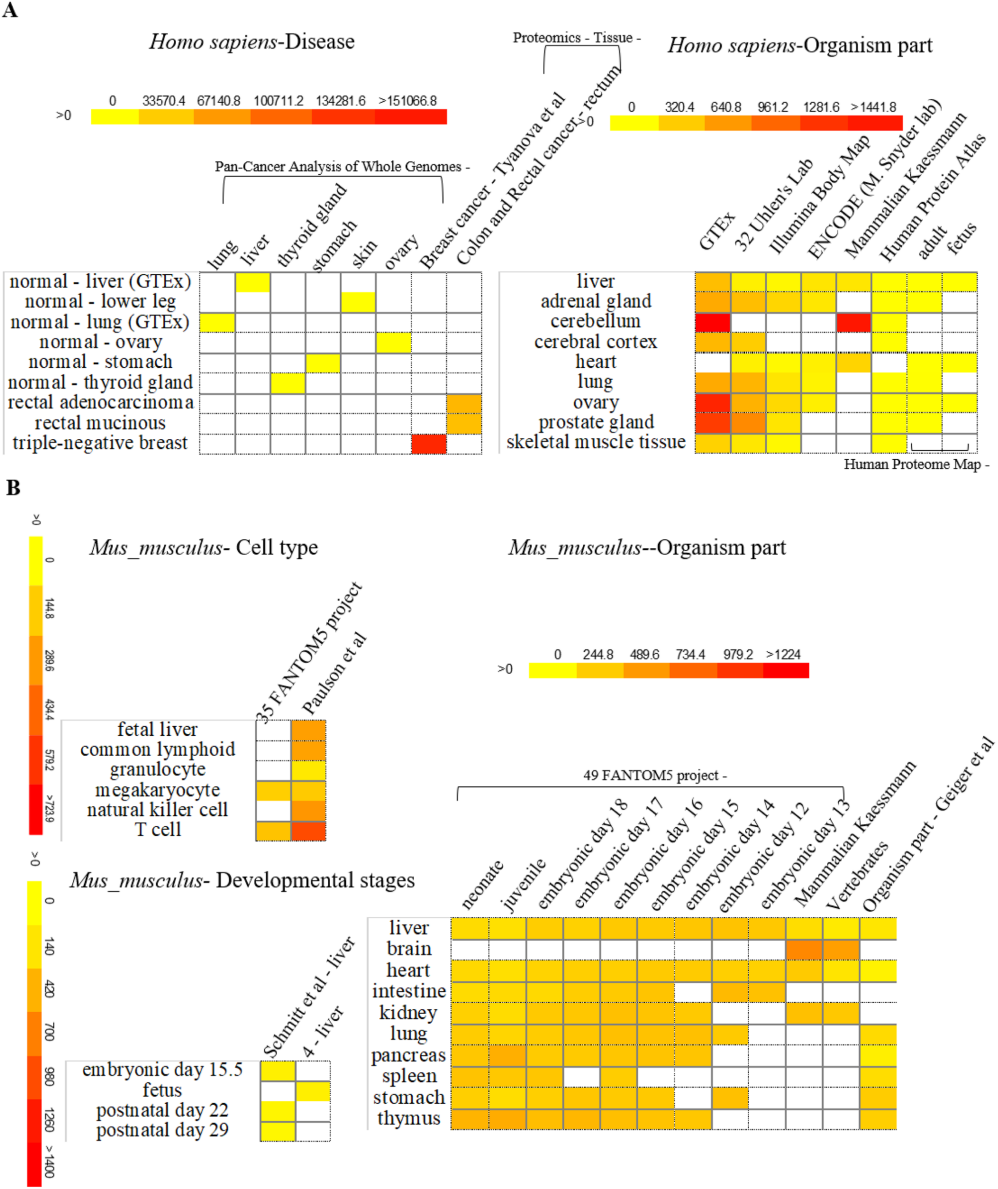
Expression of *U1-70K* in model organism *Homo sapiens* and *Mus musculus*. (**A**) Selected expression profile of human *U1-70K* gene related to human diseases and organism part is presented in heatmaps. (**B**) Representative expression profile of mouse *U1-70K* gene related to cell type, developmental stages and organism part is shown in heatmaps.

In this study, because of our expertise in digestive diseases, we pay special attention to the expression of *U1-70K* gene in the digestive system or in digestive diseases (Fig. 5). Specifically, compared to other organs, human *U1-70K* was expressed at relative level from dataset of ‘Pan-Cancer Analysis’ (Fig. 5A). In contrast, this gene was highly expressed in breast, colon and rectal cancer in the dataset of ‘Proteomics-Tissue-Colon’. Furthermore, tissue-specific expression profile from multiple datasets indicated that human *U1-70K* was enriched in cerebellum, ovary and prostate gland, with much higher expression than in liver. Transcripts of mouse *U1-70K* accumulated in T cell, higher than its expression in natural killer cells or granulocytes (Fig. 5B). However, its expression level was maintained at a low level during mouse fetus development. Similar results were obtained from multiple datasets of mouse development and tissue-specificity studies, where the abundance of *U1-70K* was not altered in various mouse digestive organs such as liver, intestine, pancreas, spleen and stomach etc.

## Discussion

It has been demonstrated that approximately 15–35% of human diseases is caused by mis-splicing or mis-assembly of spliceosomal proteins^40,41^. However, the underlying proof of how spliceosome maintains its fidelity in response to various developmental cues or stress treatments is poorly understood. Therefore, understanding the basic mechanism of spliceosomal regulation is not only the first step to decode eukaryotic splicing machinery, but also for the discovery of novel targets for clinical drug or agrochemical development^42–44^. To this end, we made a thorough comparison and phylogenetic analysis of U1-70Ks in this work to reveal their potential regulation and structural conservation among animal species.

### Assessment of phylogeny and splicing pattern suggest conserved features among animal *U1-70K*s

**Figure 6:**
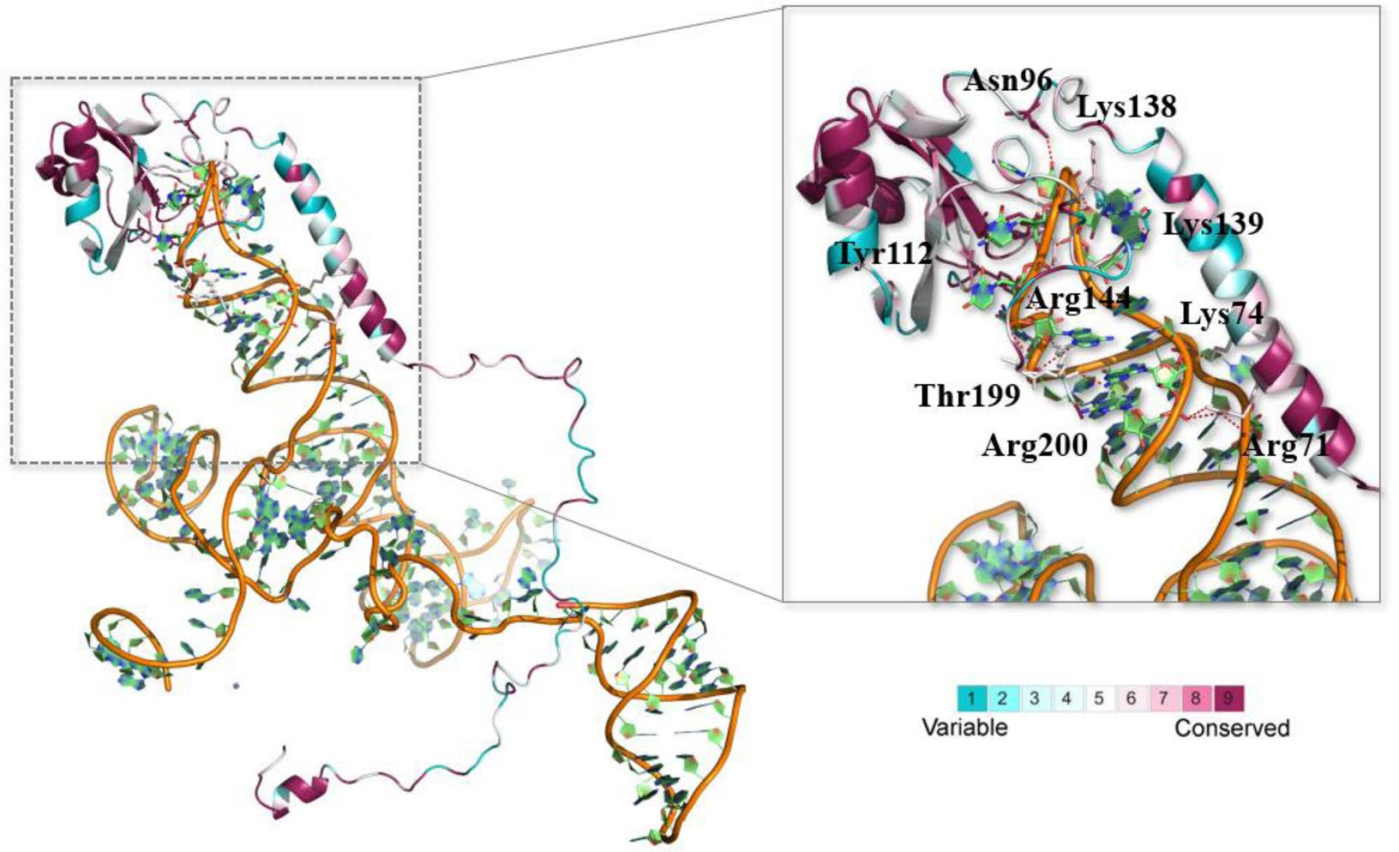
The evolutionary conservation analysis of amino acid positions in animal U1-70Ks. The crystal structure of human U1-70K (PDBID: 6QX9) with its target RNA was shown. The ribbon representation is colored according to ConSurf Grade (1-blue to 9-purple) by using all identified protein sequences of animal U1-70Ks.

The phylogenetic topology, expectedly, showed vertebrate species clustering into a large group, showing a distant relationship with other animal species such as Stramenopiles (*Aplanochytrium kerguelense*), Rhizaria (*Bigelowiella natans CCMP2755*), Alveolata (*Bigelowiella natans CCMP2755*), Amoebozoa (*Dictyostelium purpureum QSDP1*), Excavata (*Naegleria gruberi strain NEG-M*) and the outgroup of yeast, *Ciona* sequences*,* (Fig. 1, Figs. S1 and S2, left panel). Furthermore, animal U1-70Ks were subjected to conserved splicing pattern analysis (Fig. 6 and Fig. S8). Similar to plant *U1-70Ks*, animal homologs possess truncated transcripts, resulting in a conserved proteoform with C-terminal truncation (Fig. 4 and Fig. S7). However, animal U1-70Ks lack N-terminal located truncated proteoform that is conserved among plant species. Further investigation on consensus splice site sequences indicated that the C-terminal ALE event is generated by two pairs of conserved splice site sequences in vertebrates but not in other animal species and yeast, suggesting evolvement of a novel splicing mechanism specifically in vertebrates (Fig. S8). It has been suggested that non-functional isoforms are prone to be selected by negative pressure^45^, so that evolutionary conserved AS events tend to have specific biological function. However, whether these isoforms are functional in distant vertebrates needs further experimental validation. Intriguingly, we have repeatedly found conserved intron sequences in U1-70K genes lacking annotation of a second isoform, suggesting that, instead of using transcriptome data^46,47^, we may use these sequences to directly predict the existence of corresponding isoforms for genes without an isoform annotation.

### Differential expression pattern of animal *U1-70Ks* reveal their functional diversification

U1 snRNP subcomplex is critical for the assembly of early spliceosome. As a central U1 snRNP-specific protein, U1-70K connects snRNA and other U1 components during spliceosome assembly and subsequent 5′ splice site recognition. Studies have demonstrated the involvement of U1-70K in neurodegenerative diseases such as Alzheimer’s disease^23,48^, coinciding with its high expression level in cerebellum and cerebral cortex (Fig. 5). Furthermore, the knockdown of plant U1-70K partially affects fertility in the model plant Arabidopsis^49^ (Fig. 5). In addition, expression profile suggested that U1-70K may be associated with breast, rectal and colon cancers. Thus, it might help other people to design experiments to look at the roles of particular residues in protein–protein or protein-RNA interactions. Furthermore, available expression data was reported at gene level, and thus does not reflect the expression profile at isoform level. Therefore, the potential regulation of each *U1-70K* isoform might be further studied by using quantitative real-time PCR with isoform-specific primers or proteomics approaches^46,50^.

Besides of its structural divergence, the molecular function of U1-70K is conserved in animals, including its nuclear translocation ability^51^, its interaction with RNAs or other protein partners^3,52–54^, protein arginine methylation^55^ and splice site recognition mechanism^56,57^. The protein interaction network between human and mouse constructed in this analysis also supports this hypothesis (Fig. 3).

### Cross-kingdom comparison of U1-70Ks in animals, yeast and plants

**Figure 7:**
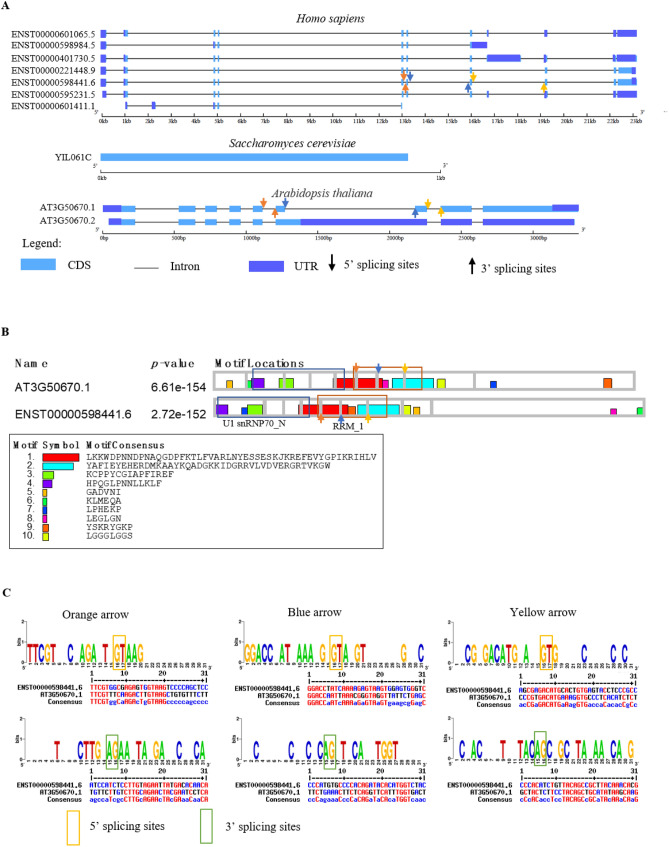
Comparison of U1-70Ks in human, yeast and Arabidopsis. (**A**) Representation of gene structures of U1-70Ks in human, yeast and Arabidopsis. Different colored arrows present different pair of splice sites. (**B**) Conserved motif analysis of human and Arabidopsis U1-70Ks. Grey boxes indicate exon–intron boundaries. (**C**) Conserved splice sites analysis of human and Arabidopsis U1-70Ks. Orange, blue and yellow arrows representing conserved splice sites are shown in left, middle and right panels, respectively. Orange boxes represent 5′ splice sites and green boxes represent 3′ splice sites.

Although splicing machinery is thought to be preserved among eukaryotes, distinct mechanisms have been observed in humans, yeast and Arabidopsis. For example, the components of U1 snRNP differ in the number of proteins and in the composition of snRNP-specific and non-specific proteins^49^. Plant U1 snRNP has been found to consist of highest number of protein members, due to several ancient duplication events in Arabidopsis lineage. Contrastingly, yeast U1 snRNP is the largest U1 subcomplex in eukaryotes due to the incorporation of yeast-specific proteins such as Prp39p and Prp40p^58^, implying its additional function during initial spliceosome assembly and splice site recognition^59^. Another interesting phenomenon is that introns from vertebrate are difficult to be excised in plant species, which may be due to mechanistic differences between animals and plants^60–62^. Previous reports indicated that human U1-70K shares approximately 44% identity to its plant counterpart^63^. We further compared genomic structure and splice site pattern of U1-70Ks from human, yeast and Arabidopsis (Fig. 7). Although, reorganization of several protein motifs was observed, U1snRNP70_N and RRM domains were preserved between human and Arabidopsis (Fig. 7B). Intriguingly, three exons encoding RRM domain of U1-70K were exactly the same between these two species (Fig. 7A,B and Table S4, highlighted). Subsequently, analysis of splice sites surrounding those three conserved exons suggested that the 3′ splice sites seems to be more conserved among eukaryotes (Fig. 7C), indicating that plants and animals may evolve similar mechanism to proof-splicing the functionally important RRM motif.

## Conclusion

In this study, we identified a total of 95 animal *U1-70K* genes and a systematic comparison of their phylogeny, genomic organization, protein and splicing conservation was performed. Animal U1-70K family genes have unique features of single copy number and a conserved splicing pattern. Given the essential role of these proteins in human disease development, understanding their biological function in animals will facilitate the development of clinical drugs or treatments.

## Supplementary Information

Supplementary Table S1.

Supplementary Table S2.

Supplementary Table S3.

Supplementary Table S4.

Supplementary Figures.
