# Supplementary material for: Phylogenetic comparison and splice site conservation of eukaryotic U1 snRNP-specific U1-70K gene family
Source: Sci Rep. 2021 Jun 17;11:12760. doi: 10.1038/s41598-021-91693-3 (PMC8211703; doi:10.1038/s41598-021-91693-3)
Supplement: Supplementary file 5 — Supplementary Figures. [file 41598_2021_91693_MOESM5_ESM.docx]

**Title:** **Phylogenetic comparison and splice site conservation of eukaryotic U1 snRNP-specific U1-70K gene family**

Running title: Phylogenetic analysis of animal *U1-70K* genes

Tao Fan^a,c,d,1^, Yu-Zhen Zhao^a,1^, Jing-Fang Yang^e,1^, Qin-Lai Liu^f,1^, Yuan Tian^c,d^, Das Debatosh^d^, Ying-Gao Liu^c^, Jianhua Zhang^g^, Chen Chen^b,2^, Mo-Xian Chen^a,2^ and Shao-Ming Zhou^a,2^

^a^ Division of Gastroenterology, Shenzhen Children’s Hospital, Shenzhen 518038, China.

^b^ Department of Infectious Disease, Nanjing Second Hospital, Nanjing University of Chinese Medicine, Nanjing 210003, P. R. China.

^c^ State Key Laboratory of Crop Biology, College of Life Science, Shandong Agricultural University, Taian, Shandong, China.

^d^ Shenzhen Research Institute, The Chinese University of Hong Kong, Shenzhen, China.

^e^ Key Laboratory of Pesticide & Chemical Biology, Ministry of Education, College of Chemistry, Central China Normal University, Wuhan 430079, P. R. China.

^f^ School of Basic Medicine, Shandong First Medical University & Shandong Academy of Medical Sciences.

^g^ Department of Biology, Hong Kong Baptist University, and State Key Laboratory of Agrobiotechnology, The Chinese University of Hong Kong, Shatin, Hong Kong.

^1^ These authors contributed equally to this work.

^2^ To whom correspondence should be addressed. Email: zhousm15d@aliyun.com, cmx2009920734@gmail.com and cindy_chenchen@126.com.

Corresponding authors

Zhou Shao-Ming and Chen Mo-Xian, Division of Gastroenterology, Shenzhen Children’s Hospital, Shenzhen 518038, China. zhousm15d@aliyun.com and cmx2009920734@gmail.com, Tel.: (86 755 81008129)

Chen Chen, Department of Infectious Disease, Nanjing Second Hospital, Nanjing University of Chinese Medicine, Nanjing 210003, P. R. China. cindy_chenchen@126.com Tel.: (86 25 85091722)


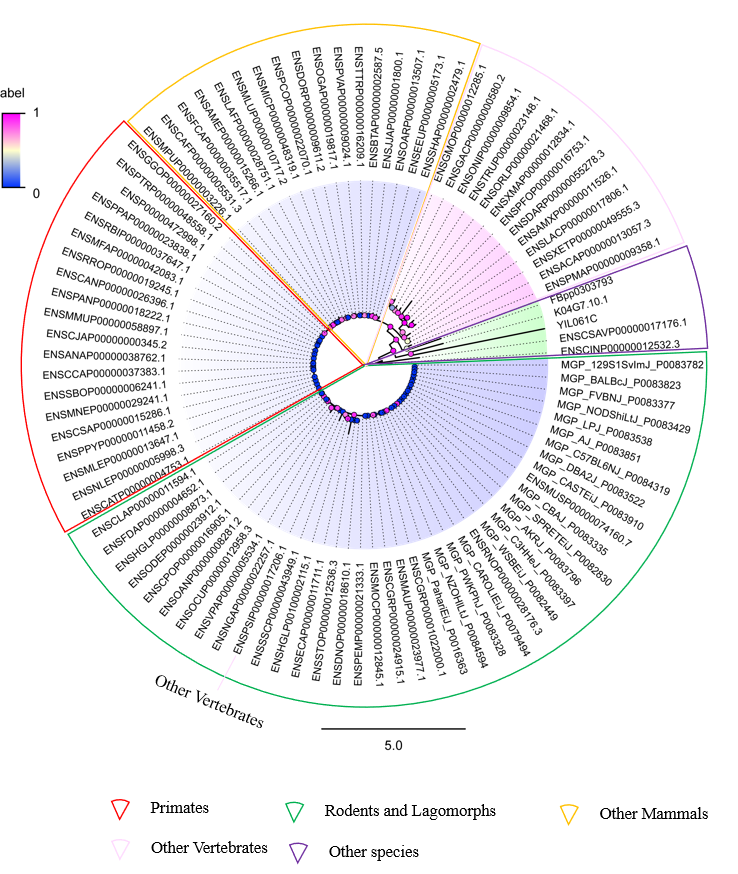


**Figure S1 Phylogenetic analysis of the *U1-70K* genes in animals.** The phylogenetic tree was constructed using maximum-likelihood method based on the amino acid sequences of 95 U1-70K members from 95 animal species/strains. The gene accession numbers from Ensembl database are displayed. Bootstrap values are presented as color gradient at each branch point. Species from different taxonomies are marked with different colors. Detailed information of each U1-70K gene was gathered in Table S1.

**
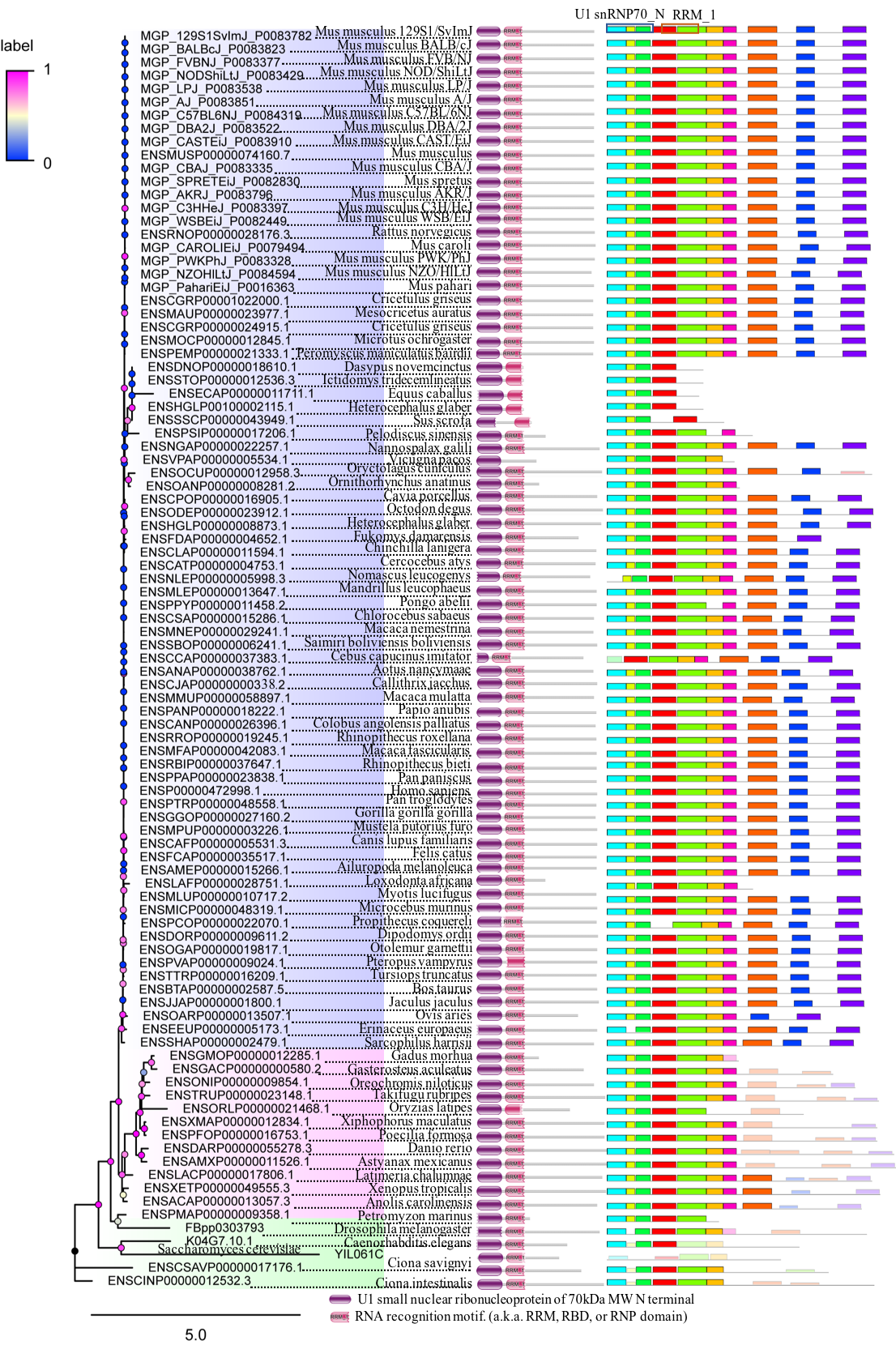
**


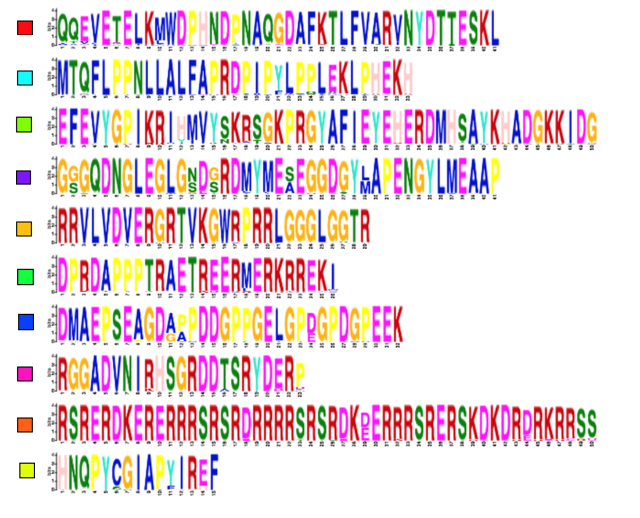

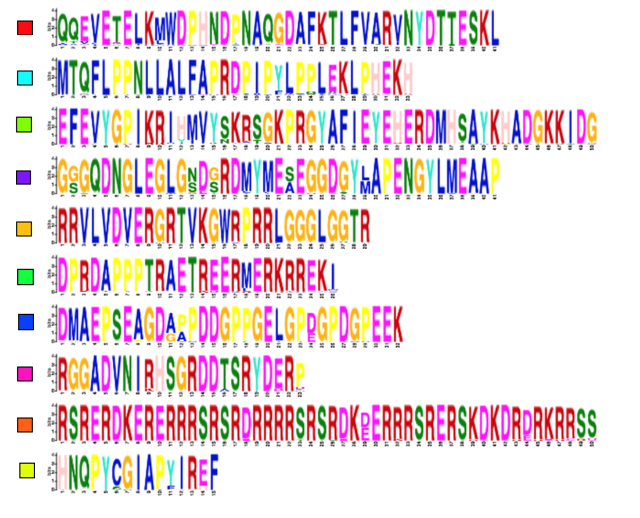


**Figure S2 Protein motif analysis of animal U1-70Ks.** The phylogenetic relationship is listed below. Protein regions predicted by online software HMMER are listed on the middle panel. Conserved motifs and sequences analyzed by MEME online tool are listed on the right panel and at the bottom of the figure, respectively. Top ten conserved motifs are represented by different colored boxes. For conserved motifs, the height of a box indicates the significance of the site (*i.e.* taller boxes are more significant). The correlation between major protein domains (middle panel) and conserved motifs (right panel) are shown in blue and red frames for U1 snRNP70_N and RRM_1 region, respectively.


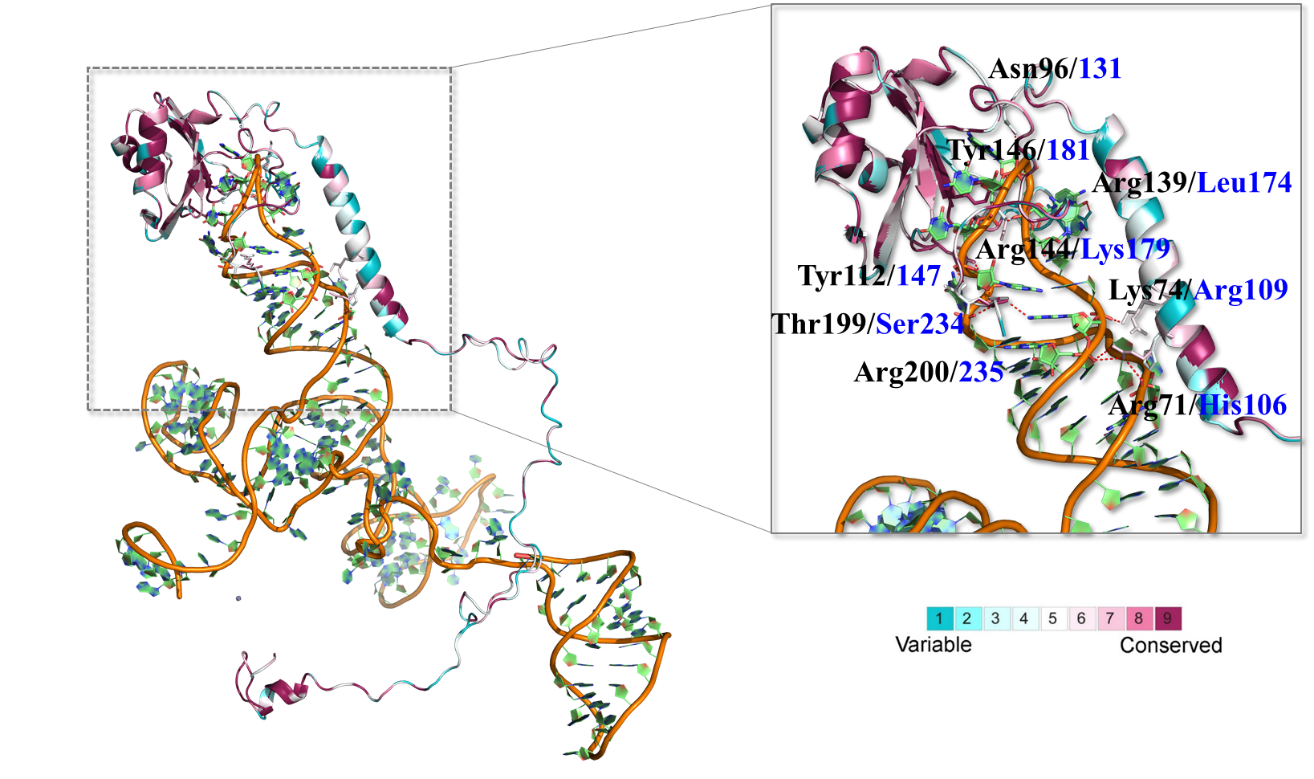


**Figure S3 Comparison of structure conservation between human and Arabidopsis U1-70Ks.** The crystal structure of human U1-70K (PDBID: 6QX9) with its target RNA was shown. The ribbon representation is colored according to ConSurf Grade (1-blue to 9-purple) by using all identified protein sequences of animal U1-70Ks. The residues names of human and *Arabidopsis thaliana* were colored in black and blue, respectively.


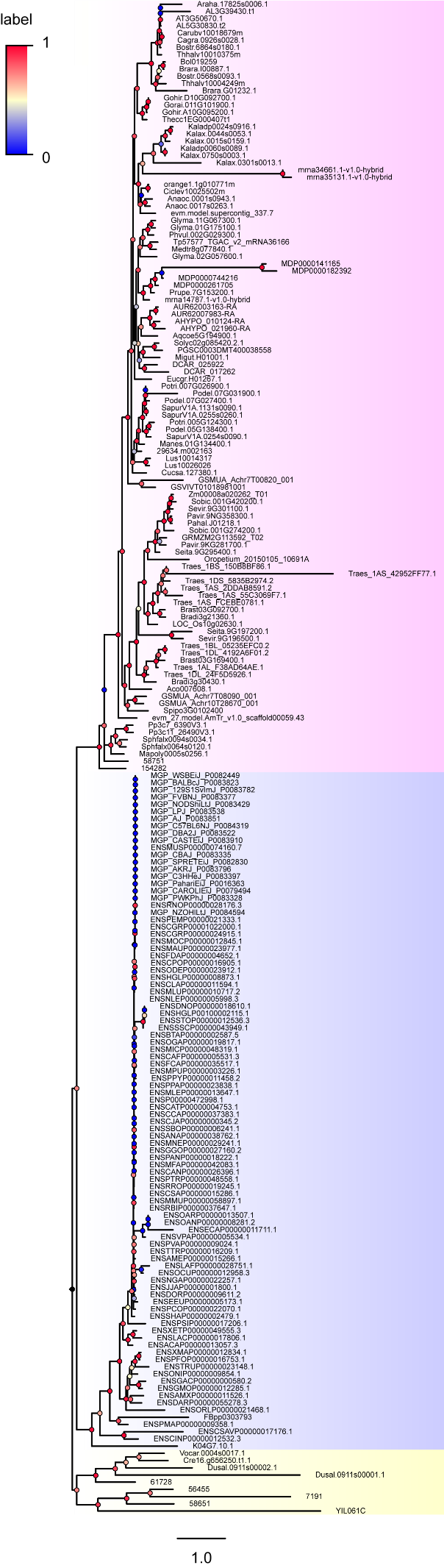


**Figure S4 Phylogenetic comparison of U1-70Ks among plants, yeast and animals.**


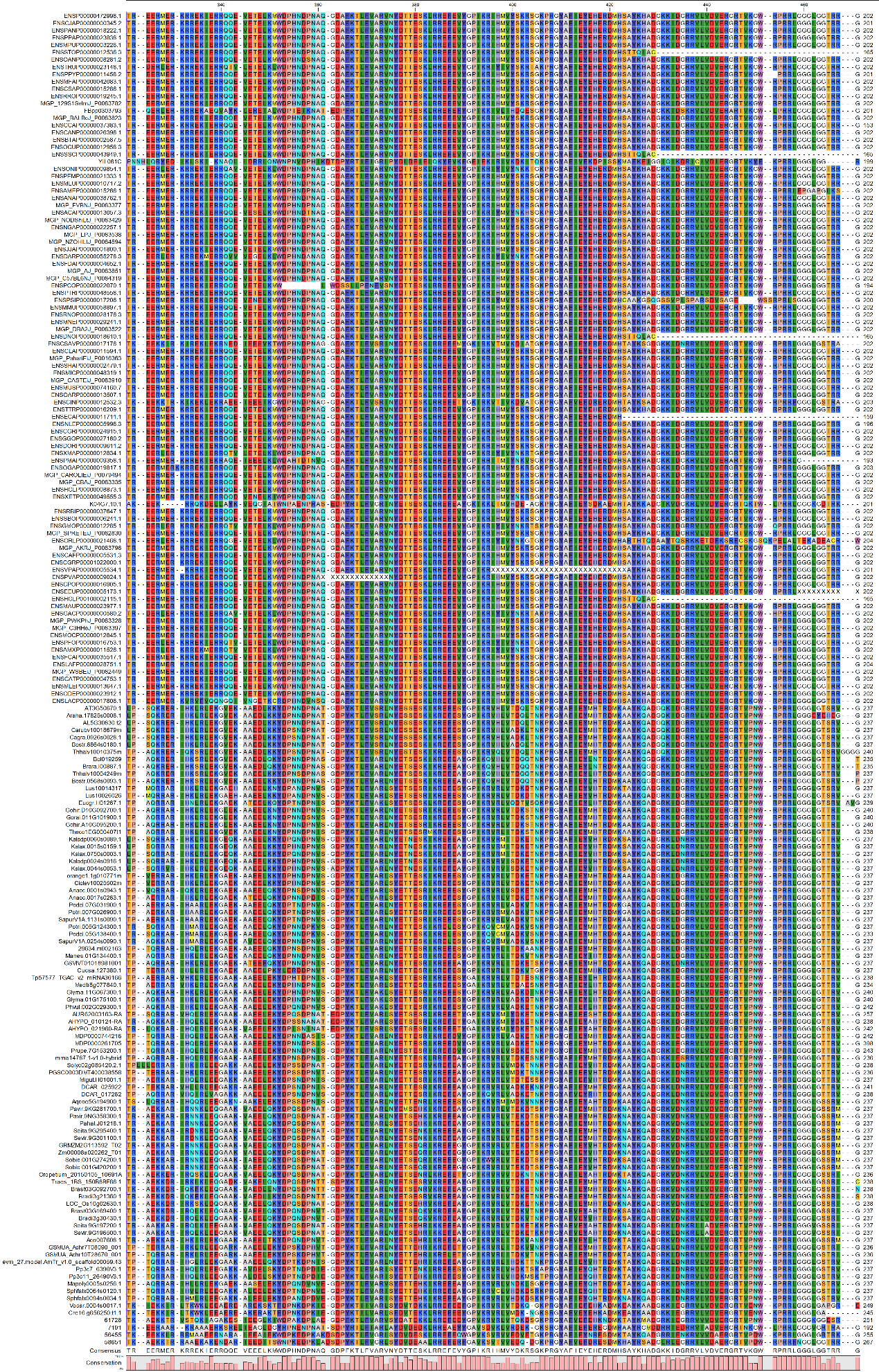


**Figure S5 Multiple sequence alignment by using both animal and plant U1-70K sequences for homology modeling.**


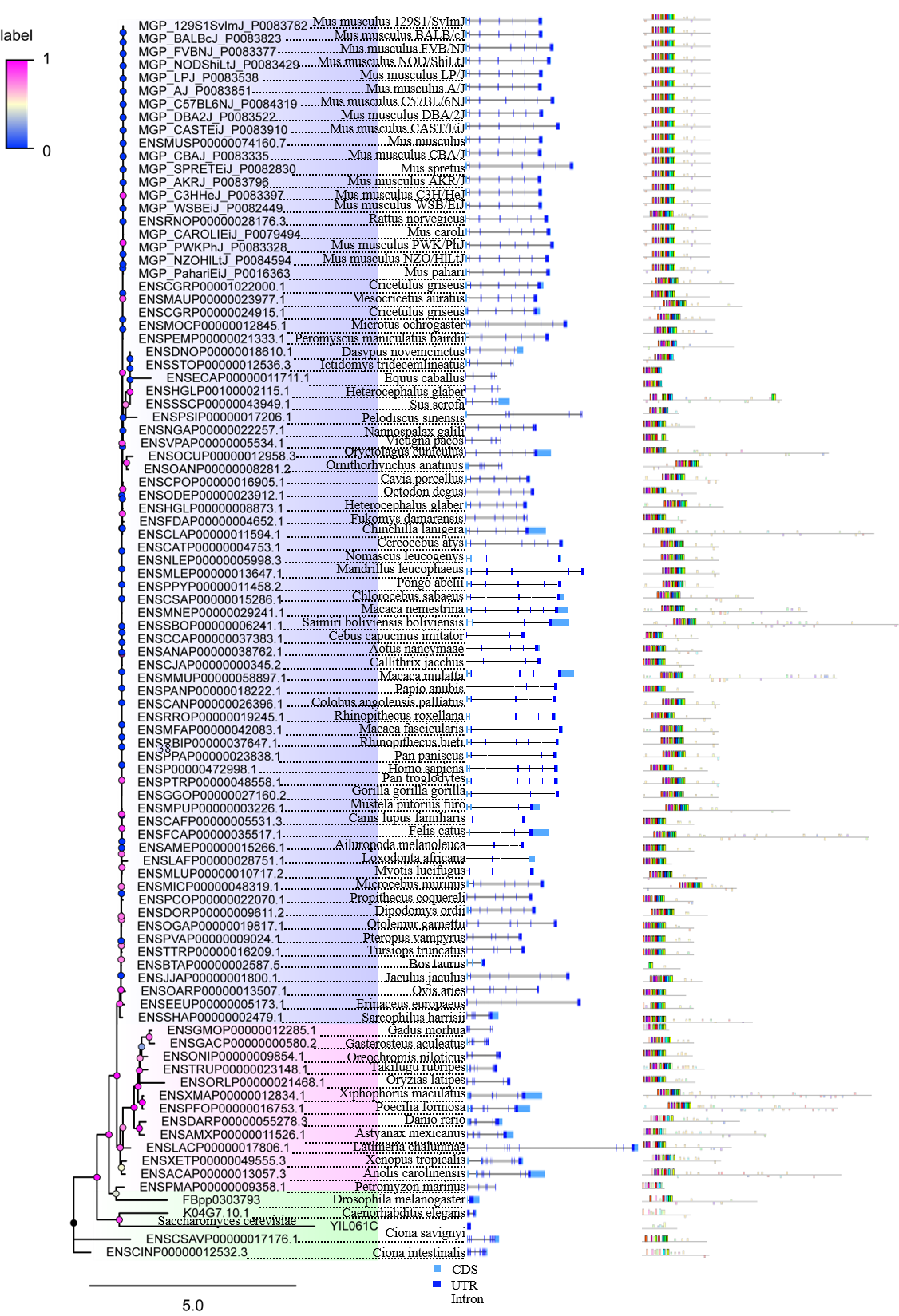


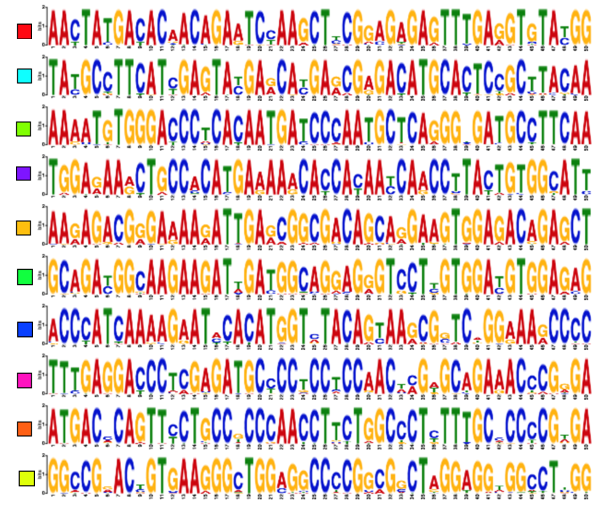

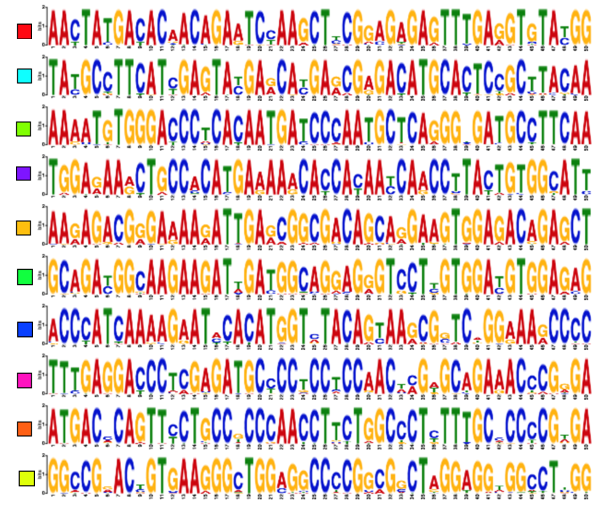


**Figure S6 Identification and comparison of genomic organization and conserved motifs among animal *U1-70K* genes.** Gene structure (middle panel) and identified cDNA conserved motifs (right panel and bottom of the figure) by MEME analysis are shown along with the vertical phylogenetic tree (left panel). The identified sequence of ten most conserved DNA motifs are listed. Different motifs are represented by different colored boxes. For conserved motifs, the height of a box indicates the significance of the site (*i.e.* taller boxes are more significant).


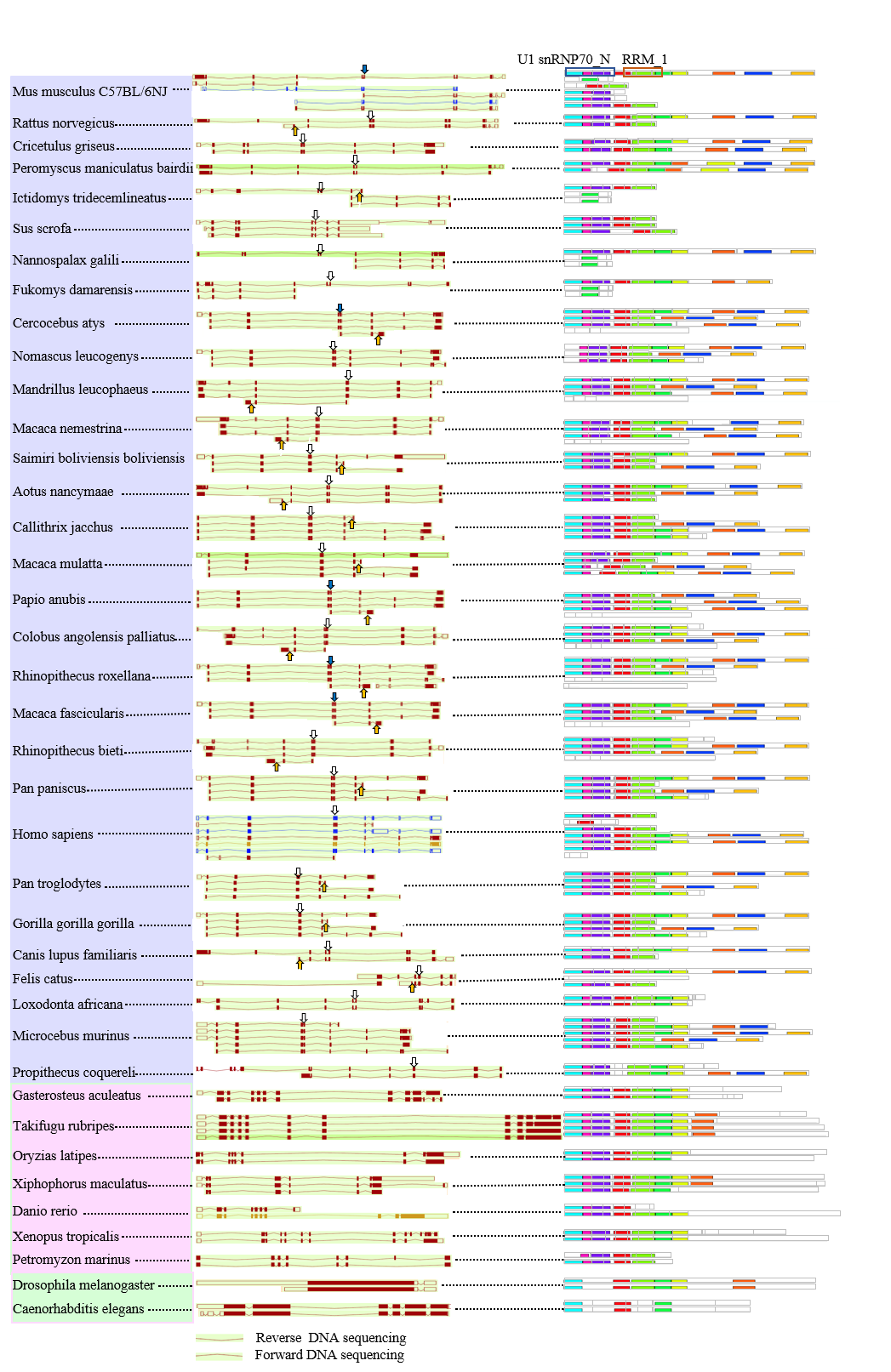


**Figure S7 Summary of splicing isoforms for animal *U1-70K* genes.** Transcript isoforms from 40 animal *U1-70K* genes are summarized (left and middle panel). Conserved protein motifs of potential protein products from splicing isoforms are illustrated (right panel) with additional annotation to define exon-exon boundaries (grey lines between boxes). Yellow arrows indicate conserved sequences found in various species. The solid and hollow blue arrows indicate the conserved splice site located in the region of RRM_1 domain with or without the detection of particular splicing events, respectively.


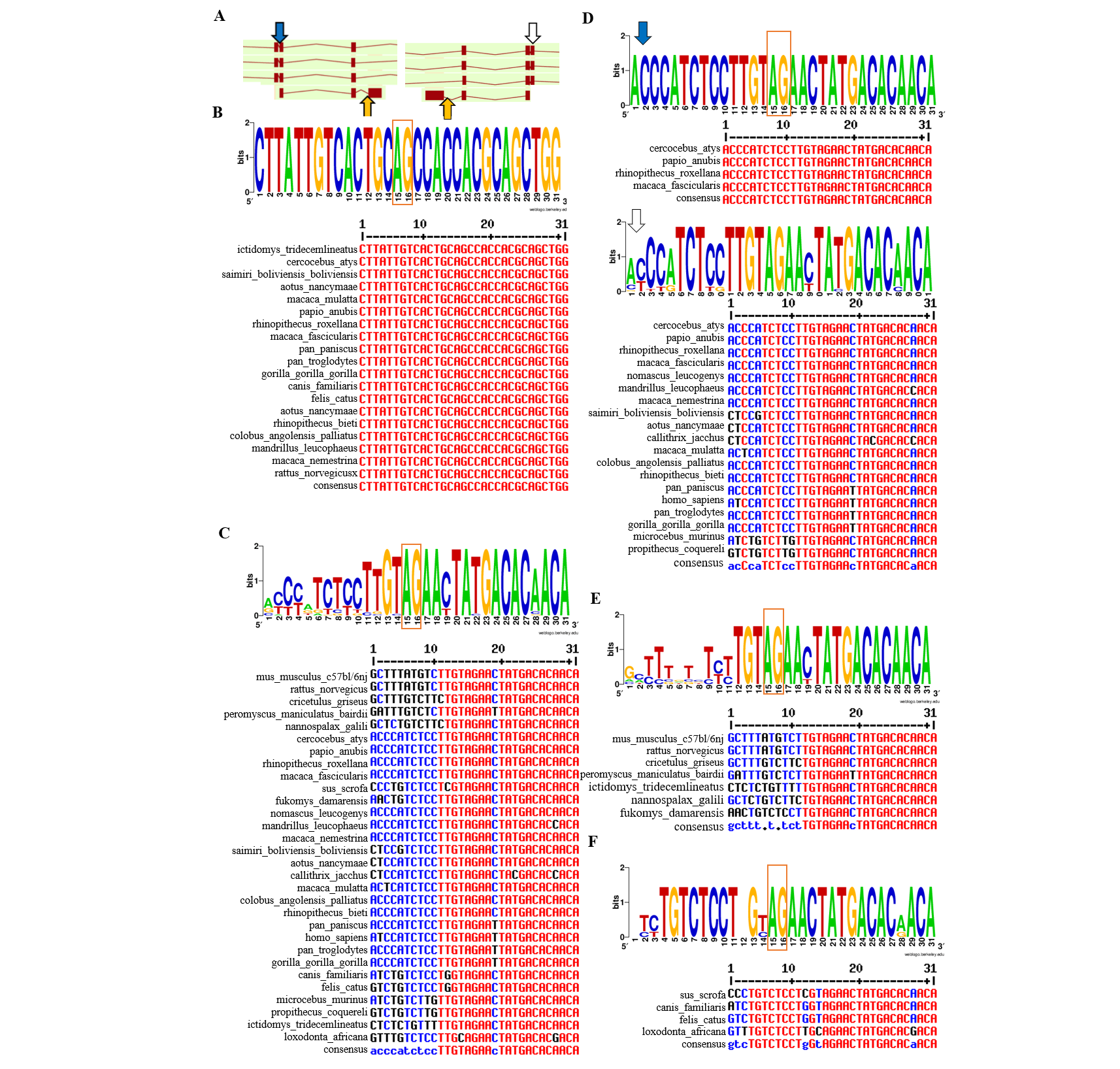


**Figure S8 Conserved alternative splice site analysis for animal *U1-70K* genes.** Flanking sequences (31-bp) at conserved exon-intron boundaries of animal *U1-70K* genes are analyzed to show their consensus by using Weblogo and multiple alignment. The conventional 3’-AG splice site dinucleotides are marked in orange boxes. **(A)** Two conserved splice sites were found among animal *U1-70K* genes as indicated by green arrow (type 1) or solid and hollow blue arrows (type 2). Graph representation and multiple alignment of type 1 **(B)** and type 2 **(C)** conserved splice sites. Detailed analysis of type 2 splice site among sector ‘primates’ **(D)**, ‘rodents and lagomorphs’ **(E)** and ‘Other Mammals’ **(F)**.


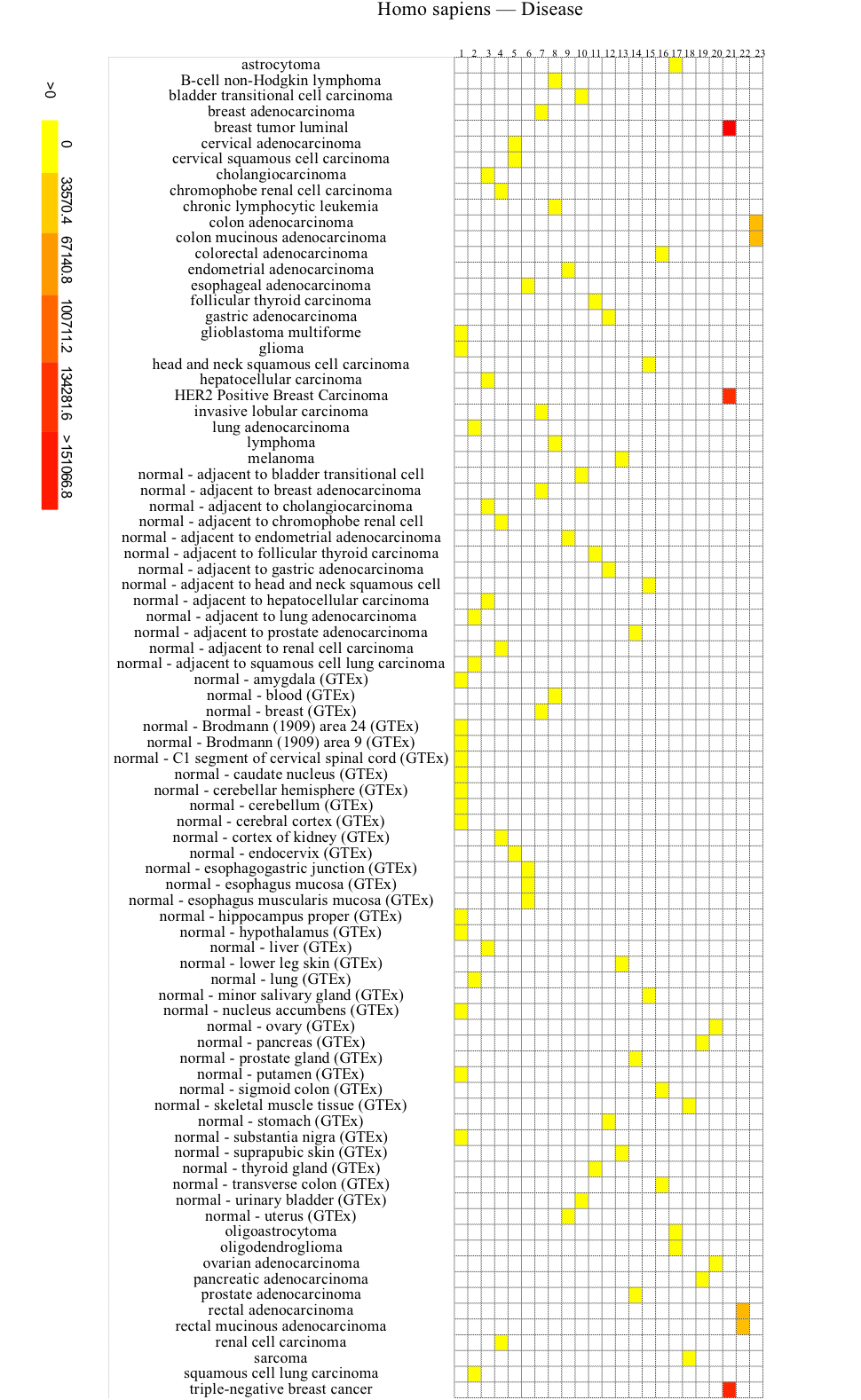


**Figure S9 Heatmap representation of disease expressions** **of *U1-70K* in *Homo sapiens*.** No.1-23 represent Pan-Cancer Analysis of Whole Genomes – brain, Pan-Cancer Analysis of Whole Genomes - lung, Pan-Cancer Analysis of Whole Genomes – liver, Pan-Cancer Analysis of Whole Genomes – kidney, Pan-Cancer Analysis of Whole Genomes - uterine cervix, Pan-Cancer Analysis of Whole Genomes – esophagus, Pan-Cancer Analysis of Whole Genomes – breast, Pan-Cancer Analysis of Whole Genomes – blood, Pan-Cancer Analysis of Whole Genomes – uterus, Pan-Cancer Analysis of Whole Genomes - urinary bladder, Pan-Cancer Analysis of Whole Genomes - thyroid gland, Pan-Cancer Analysis of Whole Genomes – stomach, Pan-Cancer Analysis of Whole Genomes – skin, Pan-Cancer Analysis of Whole Genomes - prostate gland, Pan-Cancer Analysis of Whole Genomes - mouth mucosa, Pan-Cancer Analysis of Whole Genomes - large intestine, 3 Glioma subtypes, Pan-Cancer Analysis of Whole Genomes - skeletal muscle tissue, Pan-Cancer Analysis of Whole Genomes – pancreas, Pan-Cancer Analysis of Whole Genomes – ovary, Proteomics - Tissue - Breast cancer - Tyanova et al., Proteomics - Tissue - Colon and Rectal cancer – rectum,, Proteomics - Tissue - Colon and Rectal cancer – colon, respectively. The raw data was reorganized and presented as heatmaps by using online BAR HeatMapper Plus software (http://bar.utoronto.ca/ntools/cgi-bin/ntools_heatmapper_plus.cgi)


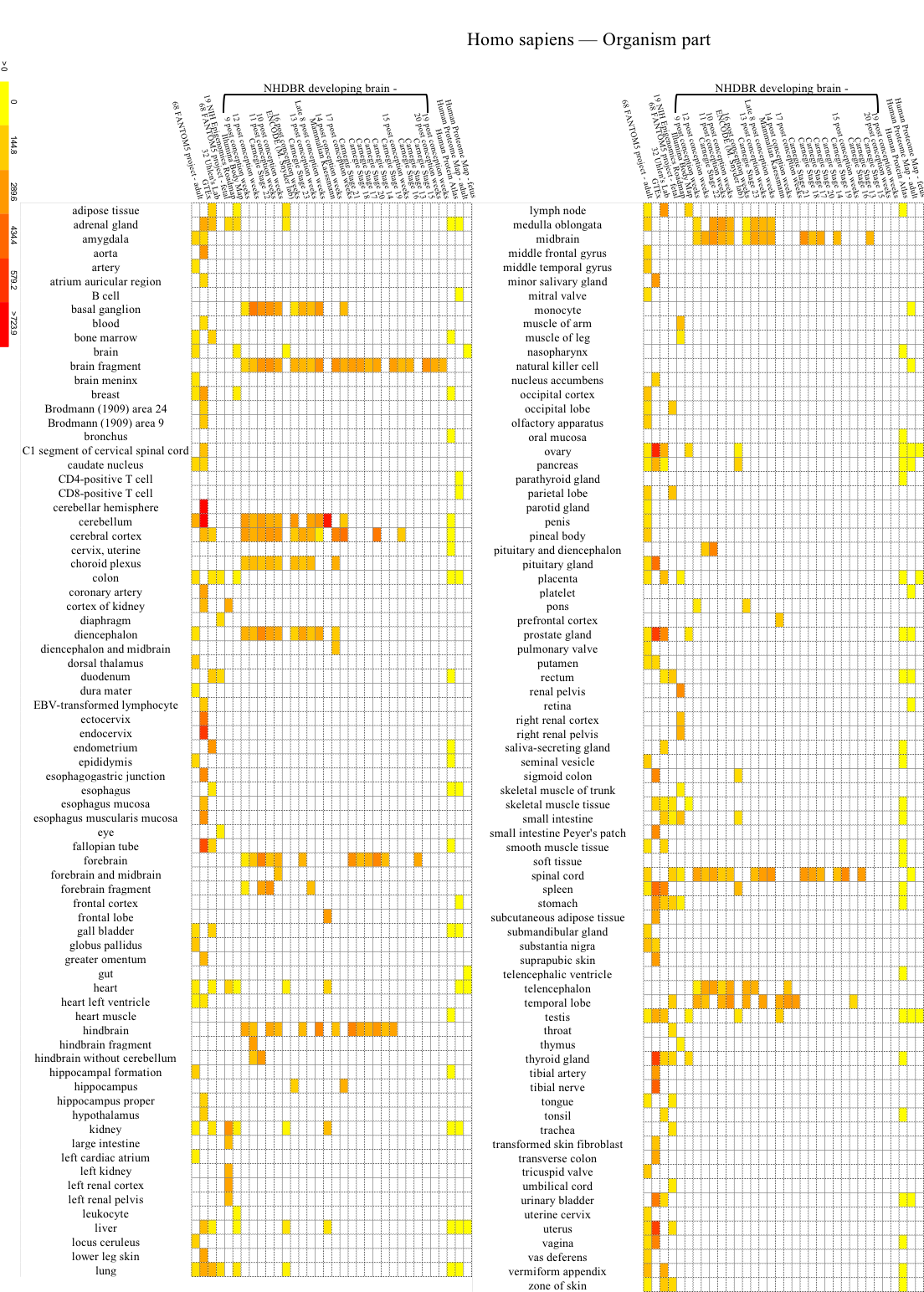


**Figure S10 Organ****-specific expressions of human *U1-70K* gene.** The raw data was reorganized and presented as heatmaps by using online BAR HeatMapper Plus software (http://bar.utoronto.ca/ntools/cgi-bin/ntools_heatmapper_plus.cgi)

**
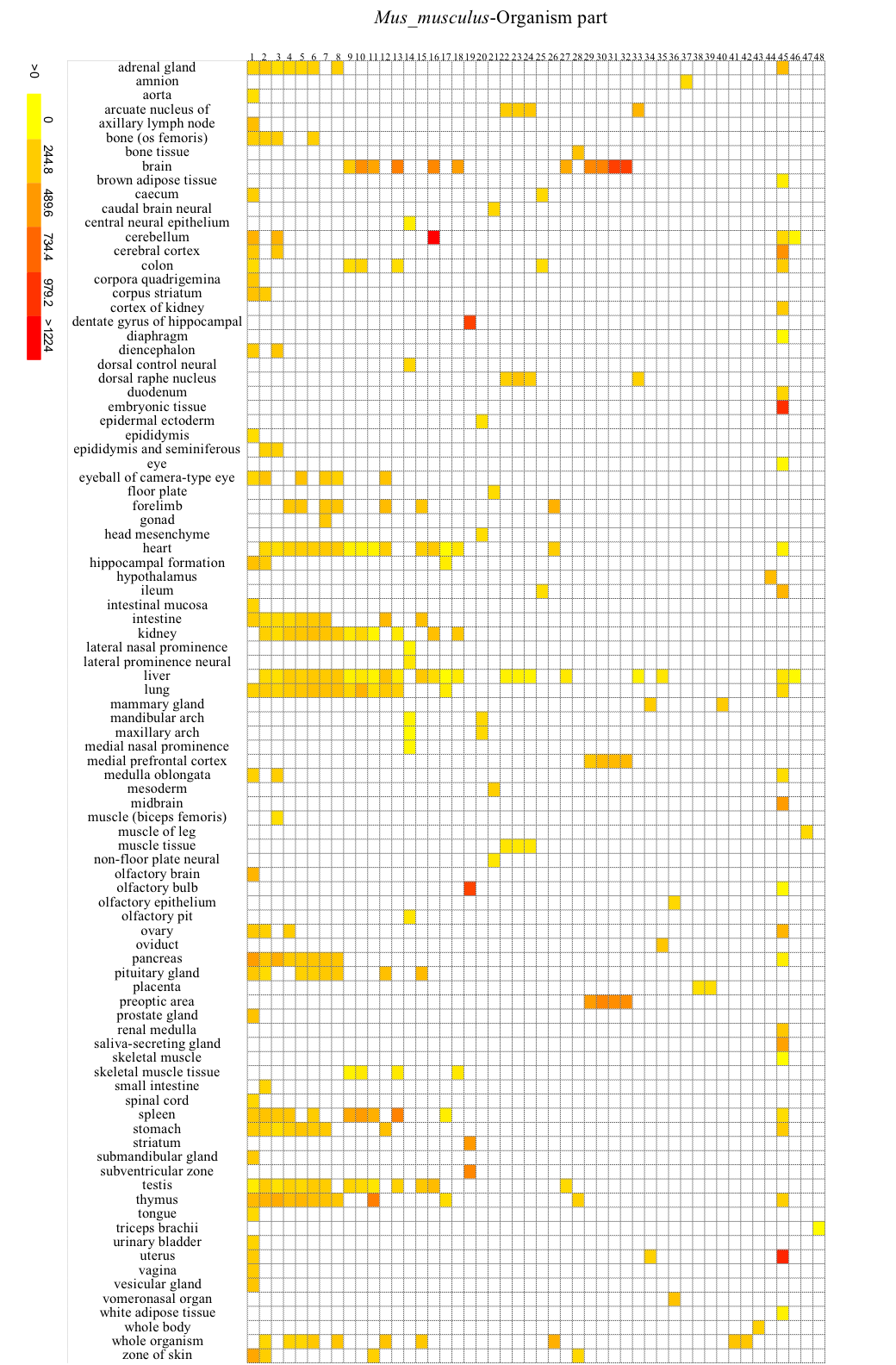
**

**Figure** **S11 Organ-specific expression of *U1-70K* in** ***Mus musculus*.** No.1-48 represents 49 FANTOM5 project - adult, 49 FANTOM5 project - neonate, 49 FANTOM5 project - juvenile, 49 FANTOM5 project - embryonic day 18, 49 FANTOM5 project - embryonic day 17, 49 FANTOM5 project - embryonic day 16, 49 FANTOM5 project - embryonic day 15, 49 FANTOM5 project - embryonic day 14, 9 in 3 strains - DBA/2J, 9 in 3 strains - CD1, 1-Sep, 49 FANTOM5 project - embryonic day 12, 9 in 3 strains - C57BL/6, 14 - embryonic day 10.5, 49 FANTOM5 project - embryonic day 13, Mammalian Kaessmann, 1-Jun, Vertebrates, 1-Apr, 14 - embryonic day 9.5, 14 - embryonic day 8.5, 4 Bonthuis et al - CastEiJ, 4 Bonthuis et al - (CastEiJ X C57BL/6J)F1, 4 Bonthuis et al - (C57BL/6J X CastEiJ)F1, Developing gut, 49 FANTOM5 project - embryonic day 11, 3 Soumillon et al, 1-Mar, Gregg et al - CAST/EiJ, Gregg et al - C57BL/6J, Gregg et al - (CAST/EiJ X C57BL/6J)F1, Gregg et al - (C57BL/6J X CAST/EiJ )F1, 4 Bonthuis et al - C57BL/6J, 49 FANTOM5 project - pregnant adult day 19, 49 FANTOM5 project - pregnant adult day 1, 1-Feb, 49 FANTOM5 project - pregnant adult day 17.5, 49 FANTOM5 project - pregnant adult day 17, 49 FANTOM5 project - pregnant adult day 10, 49 FANTOM5 project - lactating adult day 2, 49 FANTOM5 project - embryonic day 17.5, 49 FANTOM5 project - embryonic day 14.5, 49 FANTOM5 project - embryo, 4 Bonthuis et al - Idaho derived wild mouse, Organism part - Geiger et al, Organism part - Meierhofer et al, Skeletal muscle - Deshmukh et al - myotube, C2C12, Skeletal muscle - Deshmukh et al -, respectively. The raw data was reorganized and presented as heatmaps by using online BAR HeatMapper Plus software (http://bar.utoronto.ca/ntools/cgi-bin/ntools_heatmapper_plus.cgi)


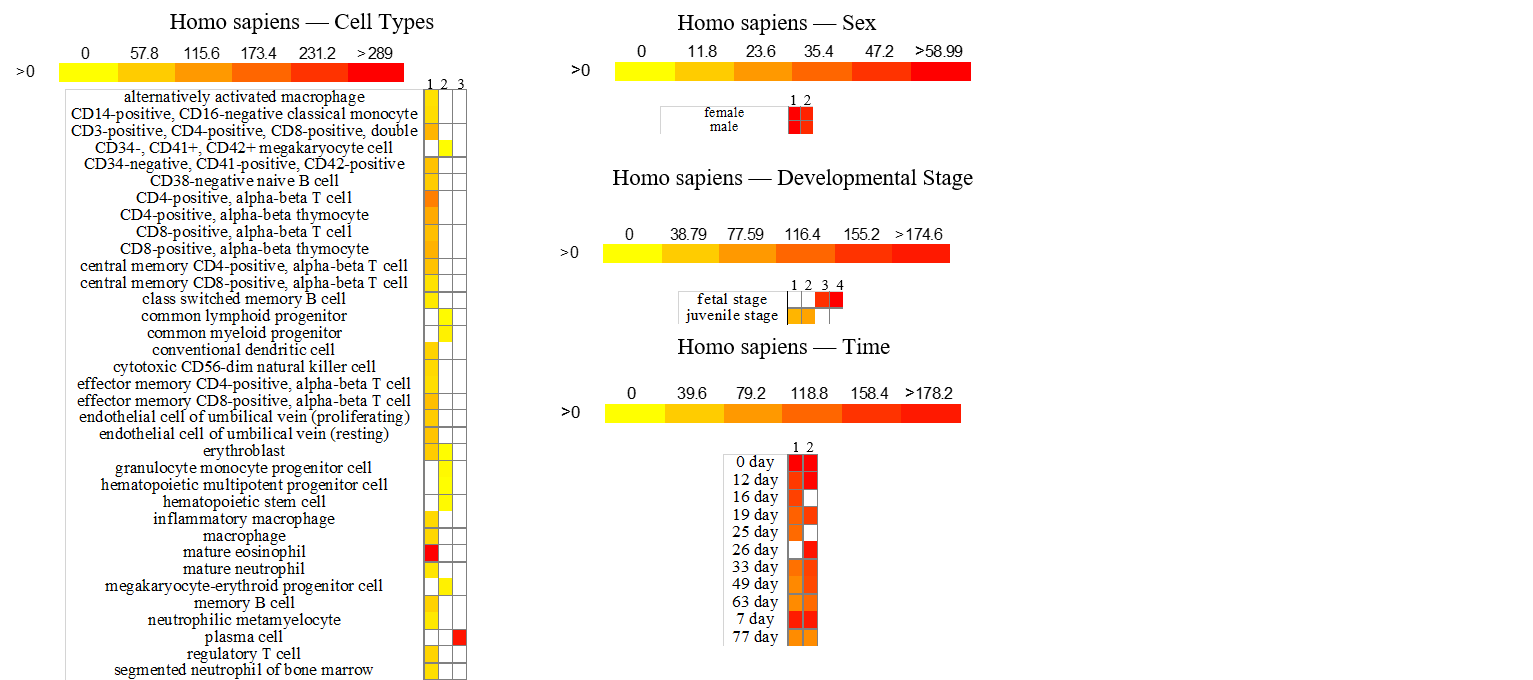


**Figure** **S12 Expression of human *U1-70K* in various** **cell types, sex, developmental stages and time****.** Homo sapiens-cell type No.1-3 mean Cell Types - BLUEPRINT common haemopoetic cells, Cell Types - BLUEPRINT rare hematopoietic cells, Cell Types - BLUEPRINT tonsil plasma cells. Homo sapiens-sex No.1 and 2 mean Sexes - Skeletal muscle, Sexes - Skeletal muscle. Homo sapiens-developmental stage No.1-4 mean Kraiczy et al intestil cells - termil ileum, Kraiczy et al intestil cells - sigmoid colon, Kraiczy et al intestil cells - proximal gut, Kraiczy et al intestil cells - distal gut. Homo sapiens-time No.1-2 mean Cortical differentiation - CORTECON - 65% confluency, Cortical differentiation - CORTECON - 90% confluency. The raw data was reorganized and presented as heatmaps by using online BAR HeatMapper Plus software (http://bar.utoronto.ca/ntools/cgi-bin/ntools_heatmapper_plus.cgi)

**
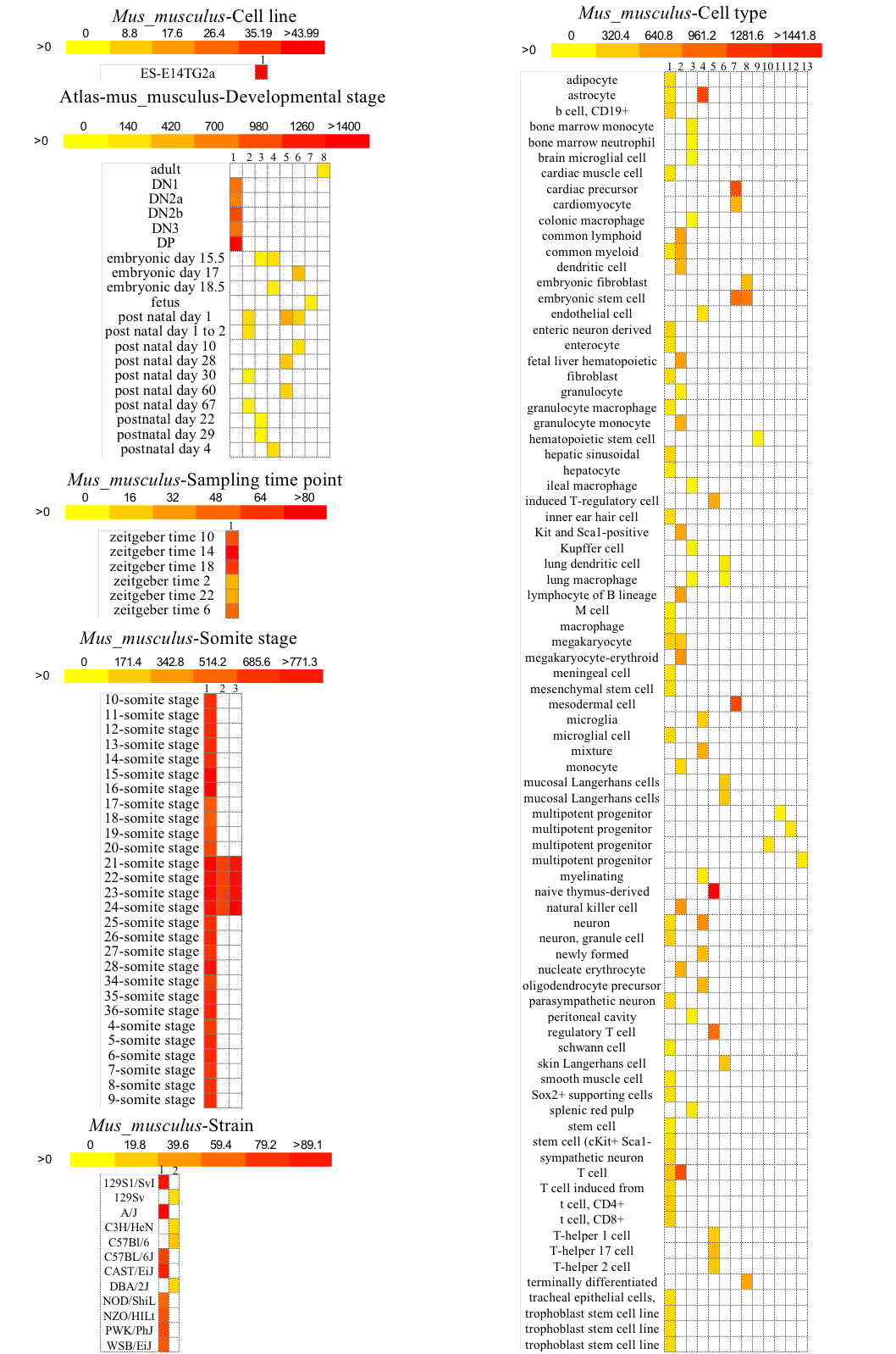
**

**Figure S13 Expression of** **mouse *U1-70K* in various** **cell lines,** **developmental stages,** **sampling time points,** **somite stages** **and strains.** *Mus musculus*-cell line 1 represents Cell line - Menschaert et al. *Mus musculus*- developmental stage No.1-8 represent 5, Cell Types - Developing heart - cardiomyocyte, Developmental stages - Schmitt et al - liver, Developmental stages - Schmitt et al - brain, Cell Types - Developing heart - fibroblast, Cell Types - Developing heart - cells of ventricle, Developmental stages - 4 - liver, Developmental stages - 4 - bone marrow.  *Mus musculus*- sampling time point 1 represents Circadian clock - Pembroke et al. *Mus musculus*- somite stage No.1-3 representwild-type embryos (DMDD), wild-type embryos (DMDD) with het parentage, pure-bred wild-type embryos (DMDD). *Mus musculus*- strain No.1 and 2 represent 8 Zheng et al, 4 Pfeiffer. *Mus_musculus*- cell type 1-13 representCell Types - 35 FANTOM5 project, Cell type - Paulson et al, Cell types - Lavin et al, Cell Types - Cerebral cortex, Cell Types - Six T cell subtypes, Cell types - Capucha et al, Cell type - Wamstad et al, Cell types - 3 Lienert et al, Cell type - Cabezas-Wallscheid et al - Lin neg (Sca-1+ c-Kit+, LSK, CD34- CD135- CD150+ CD48-), Cell type - Cabezas-Wallscheid et al - LSK (CD34+ CD135- CD150- CD48+), Cell type - Cabezas-Wallscheid et al - LSK (CD34+ CD135- CD150+ CD48-), Cell type - Cabezas-Wallscheid et al - LSK (CD34+ CD135- CD150+ CD48+), Cell type - Cabezas-Wallscheid et al - LSK (CD34+ CD135+ CD150- CD48+). The raw data was reorganized and presented as heatmaps by using online BAR HeatMapper Plus software (http://bar.utoronto.ca/ntools/cgi-bin/ntools_heatmapper_plus.cgi)
